# Status of QCD precision predictions for Drell–Yan rapidity distributions

**DOI:** 10.1140/epjc/s10052-025-14027-x

**Published:** 2025-04-10

**Authors:** S. Alekhin, S. Amoroso, L. Buonocore, A. Huss, S. Kallweit, A. Kardos, J. Michel, S. Moch, F. Petriello, L. Rottoli, Z. Trócsányi, M. Wiesemann

**Affiliations:** 1 II. Institut für Theoretische Physik, Universität Hamburg, Luruper Chaussee 149, 22761 Hamburg, Germany; 2 Deutsches Elektronen-Synchrotron DESY, Notkestr. 85, 22607 Hamburg, Germany; 3 Theoretical Physics Department, CERN, 1211 Geneva 23, Switzerland; 4 Physik Institut, Universität Zürich, 8057 Zurich, Switzerland; 5 Department of Experimental Physics, Institute of Physics, Faculty of Science and Technology, University of Debrecen, PO Box 105, Debrecen, 4010 Hungary; 6 Institute for Theoretical Physics, ELTE Eötvös Loránd University, Pázmány Péter 1/A, Budapest, 1117 Hungary; 7 Institute for Theoretical Physics Amsterdam and Delta Institute for Theoretical Physics, University of Amsterdam, Science Park 904, 1098 XH Amsterdam, The Netherlands; 8 Nikhef, Theory Group, Science Park 105, 1098 XG Amsterdam, The Netherlands; 9 Department of Physics and Astronomy, Northwestern University, Evanston, IL 60208 USA; 10 HEP Division, Argonne National Laboratory, Argonne, IL 60439 USA; 11 Dipartimento di Fisica G. Occhialini, Università degli Studi di Milano-Bicocca and INFN, Sezione di Milano-Bicocca, Piazza della Scienza, 3, 20126 Milan, Italy; 12 Max-Planck-Institut für Physik, Boltzmannstraße 8, 85748 Garching, Germany

## Abstract

We compute differential distributions for Drell–Yan processes at the LHC and the Tevatron colliders at next-to-next-to-leading order in perturbative QCD, including fiducial cuts on the decay leptons in the final state. The comparison of predictions obtained with four different codes shows excellent agreement, once linear power corrections from the fiducial cuts are included in those codes that rely on phase-space slicing subtraction schemes. For *Z*-boson production we perform a detailed study of the symmetric cuts on the transverse momenta of the decay leptons. Predictions at fixed order in perturbative QCD for those symmetric cuts, typically imposed in experiments, suffer from an instability. We show how this can be remedied by an all-order resummation of the fiducial transverse momentum spectrum, and we comment on the choice of cuts for future experimental analyses.

## Introduction

Drell–Yan lepton pair production processes are among the most important hard scattering events at the LHC. The measured final state contains only leptons. As a result, the corresponding cross sections are known experimentally very precisely. For example, the transverse momentum distribution of Drell–Yan lepton pairs reaches a precision of 0.2 % for the normalized spectra at low values of $p_T^{(\ell \ell )}$ [1, 2]. The importance of the process is shown by its frequent use in precision extraction of the parameters in the Standard Model, such as parton distribution functions (PDFs) and the strong coupling constant [3–5]. It is also used in the determination of the mass of the $W^{\pm }$-boson [6, 7]. All these measurements require a reduction of the theoretical uncertainties to match the experimental ones. The current state of the art in the theory description has advanced significantly in recent years. It has reached next-to-next-to-next-to-leading order (N$^3$LO) accuracy at fixed order in quantum chromodynamics (QCD) perturbation theory [8, 9] at the inclusive level. Transverse momentum resummation is known at the next-to-next-to-next-to-leading logarithmic (N$^3$LL) level [10–13] and even at approximate N$^4$LL [14, 15] accuracy for differential distributions in perturbation theory. At present, the fully differential calculations at N$^3$LO accuracy employ transverse momentum subtractions [16] that neglect power corrections and necessarily rely on predictions for vector boson + jet production at the N$^2$LO accuracy. Hence, the reliable computation of cross sections at N$^2$LO in QCD is a prerequisite for further developments in the field.

In Ref. [17] a subset of the present authors published a detailed comparison of the publicly available codes [16, 18–20] for $W^{\pm }$- and *Z*-boson production, including their decay. They found differences among the predictions at the NNLO level, whose size depended on the observable. The differences were estimated as similar to and sometimes even larger than the sizes of the NNLO QCD corrections themselves. This observation suggested that the neglected power corrections in transverse momentum subtraction, and other methods that rely upon phase-space slicing to regulate real emissions, could be the source of the differences. Depending on the fiducial cuts, those power corrections become linear, and hence not negligible.

The publication triggered discussions among the authors of the relevant codes, which resulted in a better understanding of the neglected terms and improvements in the computations. In this paper we provide an update of the comparisons carried out in Ref. [17]. The authors of the codes have provided new predictions for the benchmark calculations that we present in Sect. 2, now showing excellent agreement. Based on this validation of fixed-order perturbative QCD calculations through NNLO we then study the impact of fiducial cuts on the decay leptons. To that end, we focus on the case of symmetric cuts on the transverse momenta of the leptons, as they are routinely imposed in experimental analyses but display certain unphysical features [21]. In Sect. 3, we study cuts on the transverse momenta staggered in a range from a few tens of MeV to a few GeV in perturbative QCD, both at fixed order and applying all-orders resummation of large lepton-pair transverse momentum logarithms. We comment on proposed modifications of the fiducial cuts put forward recently [22]. Since the pathology observed in fixed-order perturbation theory arises from the region of small lepton-pair transverse momentum we can cure it with resummation of the small $p_T$ region. We do so in Sect. 4 and find only small differences between the resummed results and the available fixed-order codes. We provide a short discussion of the experimental resolution to gauge the impact of our findings on current analyses in Sect. 5. We summarize in Sect. 6 and conclude with our comments on the choice of cuts for experimental Drell–Yan analyses. Cross sections and information on the set-up and input parameters for some of the codes used for the benchmark comparisons are listed in the Appendices A–E.

## Benchmark computations

### Set-up and code validation

The set-up and validation for benchmarking theory predictions for $W^{\pm }$- and *Z*-boson hadro-production cross sections up to NNLO in QCD were described in Ref. [17]. In order to study the effect of cuts on the fiducial phase space in the experimental measurement two sets of data on $W^{\pm }$- and *Z*-boson production have been chosen, collected at the LHC by the ATLAS experiment and at the Tevatron by the DØ experiment, respectively.

- (Pseudo-)rapidity distributions for the decay leptons for the $W^\pm$- and $Z/\gamma ^*$-production cross sections [23] measured by the ATLAS experiment at a center-of-mass energy of $\sqrt{s}=7$ TeV, where the leptonic transverse momenta $p_T^\ell$ and pseudo-rapidities are subject to fiducial cuts.
- Distributions in the electron pseudo-rapidity for the electron charge asymmetry measured by the DØ experiment in $W^\pm$-boson production at $\sqrt{s}=1.96$ TeV at the Tevatron [24]. The DØ data is taken with fiducial cuts on the transverse momenta $p_T^{e,\nu _e}$ of the electron and the missing energy, both symmetric as well as staggered, and on their pseudo-rapidities.

The above choices are representative of the numerous data sets collected at the LHC and the Tevatron in which symmetric cuts on the final-state lepton phase space are imposed. In our theoretical predictions we use the $G_\mu$ scheme with input values $G_F$, $M_Z$, $M_W$. The QED coupling $\alpha (M_Z)$ and $\sin ^2(\theta _W)$ are then output values, which minimizes the impact of NLO electroweak corrections, see e.g. Ref. [25]. The SM input parameters are [26]

1 $$\begin{aligned} \begin{array}{ll} G_{\mu } = 1.16637\times 10^{-5} \, \textrm{GeV}^{-2} , \\ M_Z = 91.1876 \, \textrm{GeV} , \quad \Gamma _{Z} = 2.4952 \, \textrm{GeV} , \\ M_W = 80.379\, \textrm{GeV} , \quad \Gamma _W = 2.085\, \textrm{GeV} , \end{array} \end{aligned}$$

and the relevant CKM parameters are

2 $$\begin{aligned}  &   |V_{ud}| \,=\, 0.97401 , \quad |V_{us}| \,=\, 0.2265\phantom {0} , \nonumber \\  &   |V_{cd}| \,=\, 0.2265\phantom {0} , \quad |V_{cs}| \,=\, 0.97320 , \nonumber \\  &   |V_{ub}| \,=\, 0.00361 , \quad |V_{cb}| \,=\, 0.04053 . \end{aligned}$$

The computations are performed in the $\overline{\textrm{MS}}$ factorization scheme with $n_f=5$ light flavors with the $n_f=5$ flavor PDFs of ABMP16 [3, 27] as an input and the value of the strong coupling, $\alpha _s^{(n_f=5)}(M_Z) = 0.1147$. The renormalization and factorization scales $\mu _R$ and $\mu _F$ are taken to be $\mu _R = \mu _F = M_V$, with $M_V$ being the mass of the gauge boson $V = W^\pm , Z$. We note that the main results of our study are insensitive to these parameter choices.

The following codes for the computation of the fully differential NNLO QCD predictions for the lepton rapidity distributions are considered:

- DYTURBO (version 1.2) [28]^1^
- FEWZ (version 3.1) [19, 29]^2^
- MATRIX (version 2.1) [30];^3^MATRIX uses the scattering amplitudes from OpenLoops [31]. This version supersedes the previous one (version 1.0.4) [32].
- NNLOJET [33]^4^

These codes differ by the subtraction schemes used, which are either fully local or based on final-state phase space slicing in a resolution variable. FEWZ and NNLOJET employ fully local subtraction schemes, with FEWZ using sector decomposition [34] and NNLOJET using antenna subtraction [35]. NNLOJET is a Monte Carlo parton-level event generator, providing fully differential QCD predictions at NNLO for a number of LHC observables [36]. Codes based on phase space slicing methods are DYTurbo and MATRIX. DYTurbo features an improved reimplementation of the DYNNLO code [16, 18]^5^ for fast predictions for Drell–Yan processes [28]. It also includes the resummation of large logarithmic corrections. DYTurbo and MATRIX both use $q_T$-subtraction [16] at NNLO. The slicing parameters are $q_{T, \mathrm cut}$ for DYTurbo and $r_{\textrm{cut}} = q_{T,\mathrm cut}/M$ for MATRIX where *M* is the mass of the two-body final state.

Subtraction schemes based on phase space slicing are susceptible to power corrections in $q_T$. While these power corrections for vector-boson mediated process are known to be quadratic in the absence of fiducial cuts [37–41], they become linear in the presence of cuts applied on the transverse momenta of the two final state particles [17, 22, 32, 42]. The appearance of linear power corrections in the fiducial phase space is a purely kinematic effect, which allows for their efficient computation via a suitable recoil prescription [43–45].

DYTurbo (version 1.2) [28] and the new release MATRIX (version 2.1) [30] include the computation of linear power corrections in $q_T$ for $2 \rightarrow 2$ processes mediated by a vector boson. This reduces significantly the dependence of the predictions on the parameter for the $q_T$ cut-off. Additionally, MATRIX (version 2.1) also includes a bin-wise $r_{\textrm{cut}}$ extrapolation, which allows the user to obtain a yet more robust prediction than the one obtained with a finite value of $r_{\textrm{cut}}$. All the predictions shown below and obtained with MATRIX (version 2.1) include both the inclusion of linear power corrections as well as the new bin-wise extrapolation feature.

The initial benchmark [17] also considered MCFM (version 9.0) [46] and found large effects from linear power corrections in comparison to FEWZ results (local subtraction). The MCFM code^6^ implements the NNLO computation of Ref. [47] and applies *N*-jettiness subtraction [20, 48] with $\tau _{\textrm{cut}}$ as the jettiness slicing parameter. Since the recoil prescription [43–45] to remove the linear power corrections cannot be easily adapted to *N*-jettiness slicing we refer to Ref. [17] for predictions with this version of the code. As of MCFM (version 10.0) [14] the code also allows for all NNLO calculations implemented (as well as for the N$^3$LO calculations for charged- and neutral-current Drell–Yan) to be performed using $q_T$-subtraction, also accounting for fiducial power corrections, see, e.g. Refs. [49, 50].

### NNLO benchmark predictions

We computed the QCD predictions at NNLO accuracy for $W^\pm$- and $Z/\gamma ^*$-production cross sections at $\sqrt{s}=7$ TeV with the cuts imposed by ATLAS [23] using the ABMP16 PDFs [3]. The conclusions do not depend on this choice. The ATLAS data were not included in the ABMP16 PDFs fit and are shown to illustrate the accuracy of the experimental measurements. Following the set-up of the previous study [17] the predictions from FEWZ are chosen as the baseline, to which other predictions are compared.

**Fig. 1:**
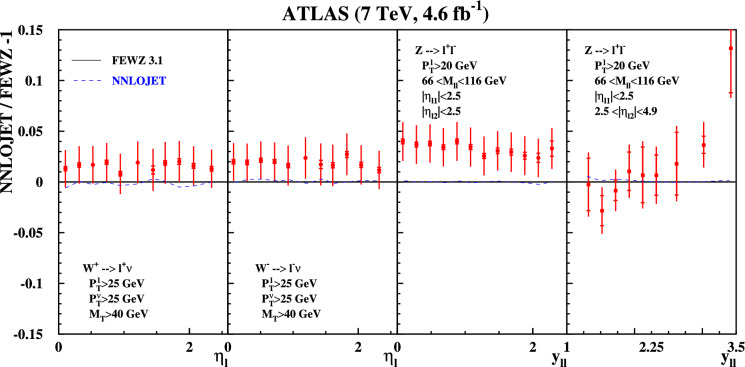
Relative deviation of ATLAS data measured in inclusive $pp \rightarrow W^\pm +X \rightarrow l^\pm \nu + X$ and $pp \rightarrow Z/\gamma ^*+X \rightarrow l^+l^- + X$ production at $\sqrt{s}=7$ TeV [23] with the statistical (inner bar) and the total uncertainties, including the systematic ones. The fiducial cuts on the decay leptons in the final state are indicated in the figure. The ABMP16 central predictions at NNLO are obtained with FEWZ and the deviations of the predictions from NNLOJET are shown (dashed) for comparison

We begin by comparing the two codes based on a local subtraction scheme, FEWZ and NNLOJET, with the 7 TeV ATLAS data. The comparison of both codes with the data as a function of lepton $p_T$ is shown in Fig. 1. We note that both codes are in excellent agreement with each other, with relative deviations between them at the per-mille level. Both codes are in reasonable agreement with the $W^{\pm }$- and *Z*-boson data, with the largest differences of 3–4% occurring for *Z*-boson production with central leptons.

**Fig. 2:**
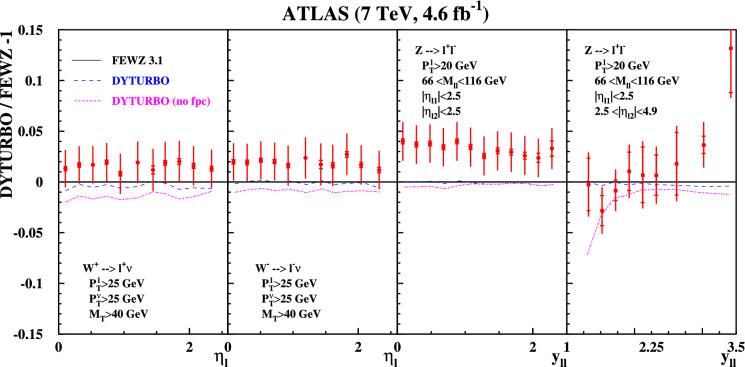
Same as Fig. 1 using predictions by the DYTURBO code with (dashed) and without (dashed-dotted) the linear power corrections labeled “(no fpc)” for comparison

**Fig. 3:**
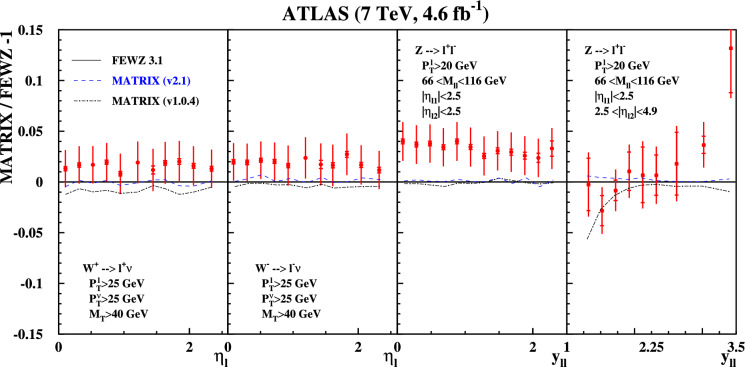
Same as Fig. 1 using predictions by the MATRIX code with version 1.0.4 (dashed-dotted) and a value for the $q_T$-slicing cut $r^{\textrm{min}}_{\textrm{cut}}=0.15\%$ as well as predictions with the improvements for the linear power corrections in version 2.1 (dashed) using $r^{\textrm{min}}_{\textrm{cut}}=0.5\%$

Having established agreement between both codes based on local subtraction schemes, as well as their agreement with the 7 TeV data, we now compare them to codes dependent on the $q_T$ slicing parameter. Figure 2 illustrates the agreement with DYTURBO (version 1.2), which accounts for linear power corrections as discussed earlier in the text. All DYTURBO predictions agree with those of FEWZ at the level of a few per-mille. The DYTURBO predictions labeled “no fpc” in Fig. 2 reproduce the DYNNLO predictions obtained previously [17], and differ from FEWZ by up to 2% for $W^{\pm }$-boson production, and by more than 5% for *Z*-boson production with forward leptons. This comparison clearly demonstrates the quality of the improvements achieved with the new version 1.2 of DYTURBO.

In Fig. 3 we show predictions for two versions of the MATRIX code normalized to the FEWZ predictions. The new MATRIX version 2.1 is distinguished by its inclusion of linear power corrections. As evident from Fig. 3 the accounting of these power corrections has a significant impact, improving the agreement with FEWZ, see also Ref. [30]. MATRIX (v2.1) agrees at the level of a few per-mille with FEWZ, with the largest deviation for *Z* production with forward leptons still significantly below 1%. The older version 1.0.4 of MATRIX without linear power corrections, considered previously [17], differs from FEWZ by 1% for $W^{\pm }$-boson production, and by up to 5% for *Z*-boson production with forward leptons.

**Fig. 4:**
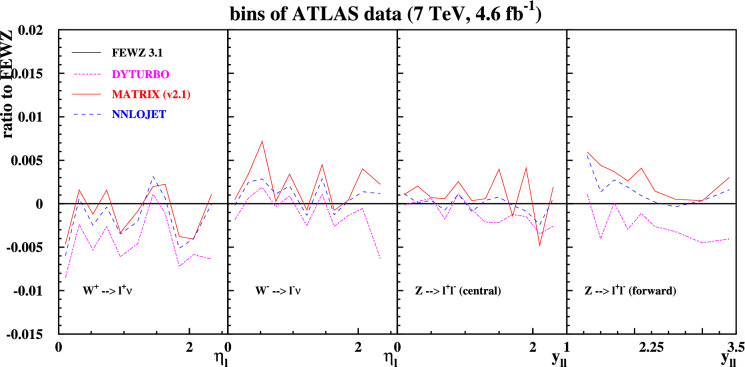
Zoom on the per-mille level agreement of the predictions from the codes DYTURBO (dashed-dotted) MATRIX (v2.1) (solid) and NNLOJET (dashed) relative to the central predictions at NNLO with FEWZ for the fiducial cuts and the bins of the ATLAS at $\sqrt{s}=7$ TeV [23]

The findings of this benchmark study are summarized Fig. 4, which compares in each case the best predictions of DYTURBO, MATRIX (v2.1) and NNLOJET. The predictions of MATRIX (v2.1) and NNLOJET are in excellent agreement, with deviations being often less than one per-mille, i.e. the target uncertainty from the numerical integration. Also the DYTURBO predictions agree very well, typically within two per-mille with the NNLOJET ones, except for the *Z*-boson production with forward leptons, where agreement is at the level of a few per-mille. Figure 4 also shows, that the predictions from DYTURBO, MATRIX (v2.1) and NNLOJET are all aligned, especially for the case of $W^{\pm }$-boson production. The normalization to the FEWZ predictions introduces some fluctuations, which are due to the numerical integration uncertainties in the FEWZ results, being at the level of a few per-mille. The predictions obtained with NNLOJET are listed in Appendix A.

**Fig. 5:**
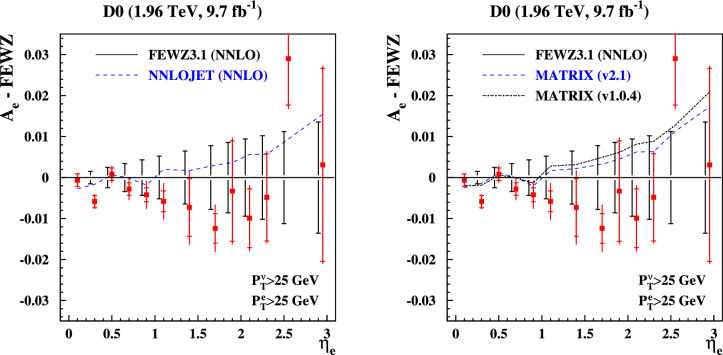
The DØ data on the electron charge asymmetry distribution $A_e$ in $W^{\pm }$-boson production at $\sqrt{s}=1.96$ TeV with the statistical (inner bar) and the total uncertainties, including the systematic ones. The difference of $A_e$ to the ABMP16 central predictions at NNLO obtained with FEWZ is shown together with the numerical integration uncertainties (black vertical solid lines) of the FEWZ predictions. The symmetric $p_T^{e,\nu }$-cuts of the decay leptons are indicated in the figure. The NNLO predictions by the NNLOJET code (dashed lines, left plot) and by the versions of the MATRIX code (dashed and dashed-dotted, right plot) are displayed for comparison

The DØ data on the electron charge asymmetry distribution $A_e$ has been obtained as a function of the electron pseudo-rapidity from $W^\pm$-boson production at $\sqrt{s}=1.96$ TeV at the Tevatron [24]. Theoretical predictions for the benchmark studies are numerically challenging due to large cancellations in the asymmetry. With predictions from NNLOJET and the update of MATRIX (version 2.1) we are in a position to check the FEWZ results, already presented in Ref. [17]. This is illustrated in Fig. 5, where we plot the NNLO predictions obtained with those codes. The numbers obtained with NNLOJET and MATRIX (version 2.1) are in excellent agreement and are also compatible with the FEWZ predictions within the substantially larger uncertainties from the numerical integration of the latter. The numerical integration uncertainties of the NNLOJET and MATRIX numbers are negligible on the scale of Fig. 5, while those of FEWZ are indicated in the plot. All numbers computed with NNLOJET are also given in Appendix A.

## Theoretical formalism for lepton fiducial cuts

To further investigate the role of lepton fiducial cuts, which lead to discrepancies between the local-subtraction codes and the slicing ones if not properly accounted for theoretically, we consider *Z*-boson production with central leptons. We stagger the $p_T$ cuts on the two leptons by a small parameter $\Delta p_T$. We review here the expected form of the cross section both in fixed-order perturbation theory and when including all-orders resummation.

### Definition of linear asymmetric fiducial cuts

Experimental analyses commonly use linear cuts on final transverse momenta. Let $p_T^{\ell _1}$ and $p_T^{\ell _2}$ be the leading and subleading lepton transverse momenta. We consider the following linear asymmetric fiducial cuts parametrized by $\Delta p_T$ (which can take either sign) and staggered on the leading and subleading leptons:

3 $$\begin{aligned}  &   p_T^{\ell _1} \ge {\left\{ \begin{array}{ll} 20 \,\text {GeV} , \quad & \Delta p_T< 0 , \\ 20 \,\text {GeV} + |\Delta p_T|, \quad & \Delta p_T> 0 ,\end{array}\right. } \nonumber \\  &   p_T^{\ell _2} \ge {\left\{ \begin{array}{ll} 20 \,\text {GeV} - |\Delta p_T|, \quad & \Delta p_T < 0 , \\ 20 \,\text {GeV} , \quad & \Delta p_T > 0 .\end{array}\right. } \end{aligned}$$

**Fig. 6:**
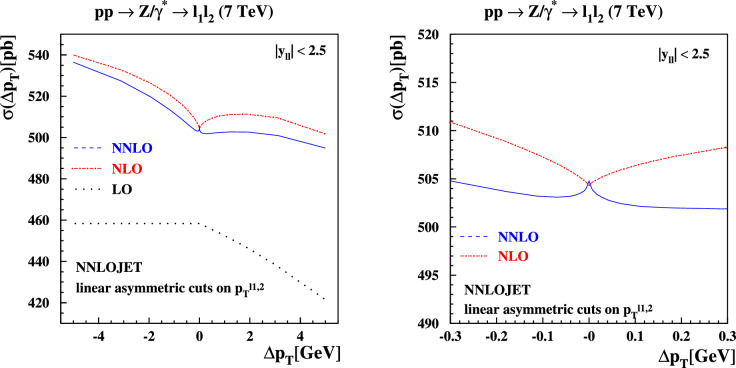
The cross sections for $pp \rightarrow Z/\gamma ^*+X \rightarrow l^+l^- + X$ production at $\sqrt{s}=7$ TeV at LO (dotted), NLO (dashed) and NNLO (solid) in QCD with ABMP16 PDFs and $y_{ll} \le 2.5$ computed with NNLOJET as a function of $\Delta p_T \in [-\,5,5]$ GeV defined in Eq. (3) for the linear asymmetric fiducial cuts on the decay leptons in the final state (left plot) and zoom on the range $\Delta p_T \in [-\,0.3,0.3]$ GeV (right plot)

**Fig. 7:**
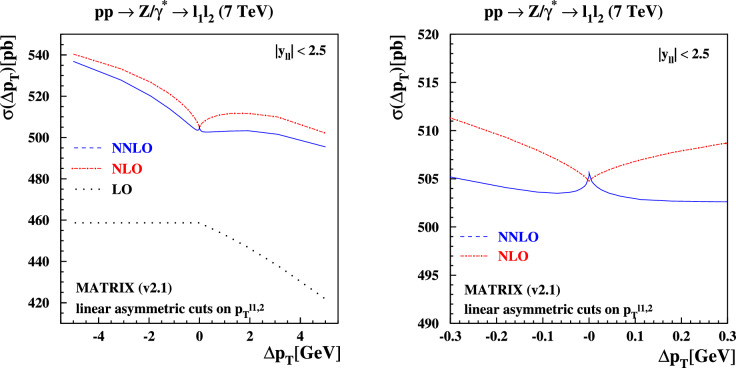
Same as Fig. 6, now showing the predictions obtained with MATRIX (v2.1)

We note that in the literature so-called staggered cuts have also been defined by applying cuts on identified leptons (electron and positron in neutral-current Drell–Yan or lepton and neutrino in charged current Drell–Yan). In this case it can be shown [17, 30, 32] that the performance of slicing methods are much improved compared to symmetric cuts or to staggered cuts imposed on leading and subleading leptons, as they lead to a quadratic sensitivity in the region $q_T < \Delta p_T$. However, we expect a similar behavior of the fixed-order cross section as a function of $\Delta p_T$ as that discussed for our definition of linear asymmetric cuts.

The phase space available in the final state decreases with increasing $\Delta p_T$, hence the cross section should monotonically decrease with this cut. We see in Figs. 6 and 7 that this occurs only at LO. A kink appears for $\Delta p_T=0$ at higher orders. The effect leading to this unphysical result was first explained in the context of photo-production of jets at HERA [21], and can be explained with the simple one-loop example presented there. Real-emission corrections collinear to the initial-state partons in the hard scattering process contain physical singularities that can be regulated with a cutoff $\delta$. Taking $\Delta p_T \ge 0$ at first, the NLO real-emission cross section $\sigma _R$ and its derivative have the following functional dependence on $\Delta p_T$ and the collinear cutoff $\delta$:

4 $$\begin{aligned} \sigma _R(\Delta p_T,\delta )= &   A(\Delta p_T,\delta )+B \, \ln \frac{\delta }{m_Z} \nonumber \\  &   \quad +C(\Delta p_T+\delta )\ln \frac{\Delta p_T+\delta }{m_Z}, \nonumber \\ \frac{d\sigma _R(\Delta p_T,\delta )}{d\delta }= &   \frac{dA}{d\delta }+\frac{B}{\delta }+C \Bigl [ 1+\ln \frac{\Delta p_T+\delta }{m_Z} \Bigr ] . \end{aligned}$$

Here, *B* and *C* are coefficients with no dependence on either $\delta$ or $\Delta p_T$, while both *A* and its derivative are regular for all parameter values. We learn two things from the above formulae. First, in the final cross section, the $B \log \delta$ term cancels against the NLO virtual diagram at this order, but still leaves behind the *C* term which is a purely real effect. Second, setting the collinear cutoff $\delta =0$ we can see that the first derivative is singular (specifically, logarithmically divergent) when $\Delta p_T \rightarrow 0$ even after adding the virtual contribution. More generally, allowing for either sign of $\Delta p_T$, including the virtual and higher-order corrections, and taking $\delta \rightarrow 0$, the fixed-order cross section at fixed $\mu _R$, $\mu _F$ and small $x = \Delta p_T/m_Z$ takes the form

5 $$\begin{aligned} \sigma (x)&= \sum _{n=0}^\infty \alpha _s^n \biggl \{ c_n + x \theta (-x) \sum _{m=0}^{2n} a_{n,m} \ln ^m (-x) \nonumber \\&\quad + x \theta (x) \sum _{m=0}^{2n} b_{n,m} \ln ^m x + {\mathcal {O}}(x^2) \,\biggr \}. \end{aligned}$$

As in the one-loop example above, the logarithmic terms are expected to arise from real emissions close to the Born configuration, i.e., a residual mis-cancellation against the corresponding virtual parts. We will make this expectation more precise in Sect. 4.

To investigate the impact of these considerations on the cross section for *Z* production at $\sqrt{s}=7$ TeV with a cut on the lepton pseudorapidities $|\eta _{\ell _1,\ell _2}| \le 2.5$ as a function of $\Delta p_T$, we present the fixed-order predictions of NNLOJET in Fig. 6 and MATRIX in Fig. 7. Results at LO, NLO and NNLO in perturbative QCD for the linear asymmetric lepton decay cuts defined in Eq. (3) are shown. Excellent agreement between the NNLOJET and the MATRIX (v2.1) numbers is observed for all values of $\Delta p_T$. The latter have been obtained accounting for the linear power corrections in the $q_T$-subtraction and bin-wise $r_{\textrm{cut}}$ extrapolation, as discussed previously. The logarithmic divergences described above lead to the kink at $\Delta p_T =0$ and the resulting non-monotonic behavior as $\Delta p_T$ is varied in these plots. This result is a pathology of fixed-order perturbation theory when symmetric cuts are applied.

### Structure of the physical cross section

It is useful to discuss the general form that the *physical* cross section should take as a function of $x = \Delta p_T/m_Z$, ignoring QED and weak corrections (but assuming that Nature exactly solved QCD for us). In this case we expect that the distribution $d \sigma /d \Phi _{\ell \ell } \equiv d^6 \sigma /(d^3 \vec {p}_{\ell ^+} \, d^3 \vec {p}_{\ell ^-})$ in the lab frame is non-negative and smooth in all limits, unlike the fixed-order cross section with its singular ridge along the surface $\vec {p}_{T,\ell ^+} = - \vec {p}_{T,\ell ^-}$. However, even in the case of the physical cross section we still generically find a discontinuous derivative as $x \rightarrow 0$ from above vs. from below. Letting $p_T^\textrm{cut} = 20 \,\text {GeV}$, this can be seen as follows:

6 $$\begin{aligned}&\frac{1}{m_Z} \frac{d \sigma (x > 0)}{d x}\nonumber \\&\quad = -\int \! d \Phi _{\ell \ell } \, \frac{d \sigma }{d \Phi _{\ell \ell }} \, \delta \Big (p_T^{\ell _1} - p_T^\textrm{cut} - x m_Z\Big ) \, \Theta \Big (p_T^{\ell _2} - p_T^\textrm{cut}\Big ) \nonumber \\&\quad = - \int \! d \Phi _{\ell \ell } \, \frac{d \sigma }{d \Phi _{\ell \ell }} \, \delta \Big (p_T^{\ell _1} - p_T^\textrm{cut}\Big ) \, \Theta \Big (p_T^{\ell _2} - p_T^\textrm{cut}\Big ) + {\mathcal {O}}(x) , \end{aligned}$$

7 $$\begin{aligned}&\frac{1}{m_Z} \frac{d \sigma (x < 0)}{d x}\nonumber \\&\quad = -\int \! d \Phi _{\ell \ell } \, \frac{d \sigma }{d \Phi _{\ell \ell }} \, \Theta \Big (p_T^{\ell _1} - p_T^\textrm{cut}\Big ) \, \delta \Big (p_T^{\ell _2} - p_T^\textrm{cut} - x m_Z\Big ) \nonumber \\&\quad = -\int \! d \Phi _{\ell \ell } \, \frac{d \sigma }{d \Phi _{\ell \ell }} \, \Theta \Big (p_T^{\ell _1} - p_T^\textrm{cut}\Big ) \, \delta \Big (p_T^{\ell _2} - p_T^\textrm{cut}\Big ) + {\mathcal {O}}(x) ,\end{aligned}$$

where $p_T^{\ell _1} = \max \Big \{ p_T^{\ell ^+}, p_T^{\ell ^-}\Big \}$ and $p_T^{\ell _2} = \min \Big \{ p_T^{\ell ^+}, p_T^{\ell ^-}\Big \}$ are functions of $\Phi _{\ell \ell }$. We see that the derivatives cannot be positive in either case, since in each case the integrand is non-negative. However, the cross sections in Eqs. (6) and (7) do not coincide. For $x > 0$ we have

8 $$\begin{aligned}&\delta (p_T^{\ell _1} - p_T^\textrm{cut}\Big ) \, \Theta \Big (p_T^{\ell _2} - p_T^\textrm{cut}\Big )\nonumber \\&\quad = \Theta \Big (p_T^{\ell _-} - p_T^{\ell _+}\Big ) \, \delta \Big (p_T^{\ell _-} - p_T^\textrm{cut}\Big ) \, \Theta \Big (p_T^{\ell _+} - p_T^\textrm{cut}\Big ) \nonumber \\  &\qquad + \Theta \Big (p_T^{\ell _+} - p_T^{\ell _-}\Big ) \, \delta \Big (p_T^{\ell _+} - p_T^\textrm{cut}\Big ) \, \Theta \Big (p_T^{\ell _-} - p_T^\textrm{cut}\Big ) \nonumber \\&\quad = \Theta \Big (p_T^\textrm{cut} - p_T^{\ell _+}\Big ) \, \delta \Big (p_T^{\ell _-} - p_T^\textrm{cut}\Big ) \, \Theta \Big (p_T^{\ell _+} - p_T^\textrm{cut}\Big ) \nonumber \\  &\qquad + \Theta \Big (p_T^\textrm{cut} - p_T^{\ell _-}\Big ) \, \delta \Big (p_T^{\ell _+} - p_T^\textrm{cut}\Big ) \, \Theta \Big (p_T^{\ell _-} - p_T^\textrm{cut}) = 0 ,\end{aligned}$$

which implies

9 $$\begin{aligned} \frac{1}{m_Z} \frac{d \sigma (x > 0)}{d x} = 0 + {\mathcal {O}}(x) , \end{aligned}$$

because the integration region is a lower-dimensional manifold (assuming some precise limit-taking treatment of $\theta (y) \theta (-y)$), whereas the physical cross section is free of distributional terms and bounded on every subdomain such that we can drop this null set. By contrast, for $x < 0$ we find a genuinely 5-dimensional integration region that is not a null set,

10 $$\begin{aligned}&\Theta \Big (p_T^{\ell _1} - p_T^\textrm{cut}\Big ) \, \delta \Big (p_T^{\ell _2} - p_T^\textrm{cut}\Big )\nonumber \\&\quad = \Theta \Big (p_T^{\ell _-} - p_T^{\ell _+}\Big ) \, \Theta \Big (p_T^{\ell _-} - p_T^\textrm{cut}\Big ) \, \delta \Big (p_T^{\ell _+} - p_T^\textrm{cut}\Big ) \nonumber \\  &\qquad + \Theta \Big (p_T^{\ell _+} - p_T^{\ell _-}\Big ) \, \Theta \Big (p_T^{\ell _+} - p_T^\textrm{cut}\Big ) \, \delta \Big (p_T^{\ell _-} - p_T^\textrm{cut}\Big ) \nonumber \\&\quad = \Theta \Big (p_T^{\ell _-} - p_T^\textrm{cut}\Big ) \, \delta \Big (p_T^{\ell _+} - p_T^\textrm{cut}\Big ) \nonumber \\  &\qquad + \Theta \Big (p_T^{\ell _+} - p_T^\textrm{cut}\Big ) \, \delta \Big (p_T^{\ell _-} - p_T^\textrm{cut}\Big ) .\end{aligned}$$

Thus, we find a non-vanishing derivative at $x < 0$,

11 $$\begin{aligned} \frac{1}{m_Z} \frac{d \sigma (x < 0)}{d x}&= - \left[ \frac{d \sigma _\textrm{fid}}{d p_T^{\ell ^-}} + \frac{d \sigma _\textrm{fid}}{d p_T^{\ell ^+}} \right] _{p_T = p_T^\textrm{cut}} + {\mathcal {O}}(x) ,\end{aligned}$$

which we can identify as the sum over the lepton $p_T$ spectra evaluated at $p_T = p_T^\textrm{cut}$, applying a fiducial $p_T \ge p_T^\textrm{cut}$ on the respective other one. Importantly, the derivative is $\le 0$, as expected because the physical fiducial cross section must decrease with $\Delta p_T$.

It is useful to note how a fixed-order calculation of $d \sigma /d \Phi _{\ell \ell }$ modifies the above conclusions. Specifically, unlike the physical cross section,

1. the fixed-order calculation is not guaranteed to be positive, so the unphysical scenario of a cross section *increasing* with $\Delta p_T$ (as the phase space is being constrained further) is possible; and
2. the fixed-order calculation generically contains terms with a finite integral but supported on a null set, which is easiest to see from the tree-level $\delta ^{(2)}(\vec {p}_{T}^{\,\,\ell ^-} - \vec {p}_{T}^{\,\,\ell ^+})$, and thus the derivative in the limit $x\rightarrow 0^+$ need not vanish.

Both of these properties are addressed by resummation at the level of the hadronic structure functions, and we thus expect matched predictions to have the physical properties derived above, which will be addressed in Sect. 4.

### Definition of product cuts

**Fig. 8:**
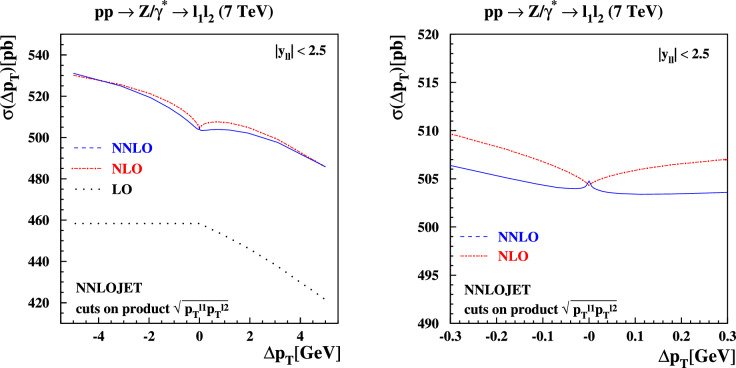
Same as in Fig. 6, now with product cuts on the transverse momenta of the decay leptons in the final state. The product cuts are defined in Eq. (12) and ranges $\Delta p_T \in [-\,5,5]$ GeV (left plot) and $\Delta p_T \in [-0.3,0.3]$ GeV (right plot) are shown

**Fig. 9:**
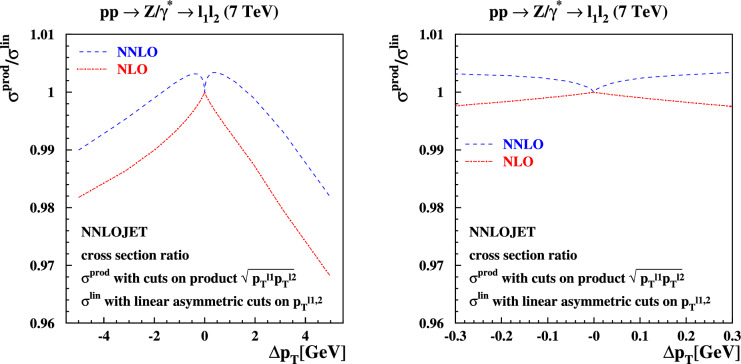
The ratio of cross sections for $pp \rightarrow Z/\gamma ^*+X \rightarrow l^+l^- + X$ production from Fig. 8 with the product cuts of Eq. (12) over those from Fig. 6 with linear asymmetric cuts of Eq. (3) as a function of $\Delta p_T$ at NLO (dashed) and NNLO (solid) in QCD. The ranges $\Delta p_T \in [-\,5,5]$ GeV (left plot) and $\Delta p_T \in [-0.3,0.3]$ GeV (right plot) are shown

Several resolutions to the problem of symmetric cuts have been suggested in the literature. In Ref. [22], the origin of this behavior was traced back to terms in the perturbative expansion of the cross section linear in the transverse momentum $p_{T}$ of the decaying *Z*-boson that produces the lepton pair. It was suggested there to replace the cuts on the separate leptons with a cut on the product of the transverse momenta of the leptons, which we implement as follows:

12 $$\begin{aligned}  &   \sqrt{p_T^{\ell _1} p_T^{\ell _2}} \ge {\left\{ \begin{array}{ll} 20 \,\text {GeV} , \quad & \Delta p_T< 0 , \\ 20 \,\text {GeV} + |\Delta p_T|, \quad & \Delta p_T> 0 ,\end{array}\right. } \nonumber \\  &   p_T^{\ell _2} \ge {\left\{ \begin{array}{ll} 20 \,\text {GeV} - |\Delta p_T|, \quad & \Delta p_T < 0 , \\ 20 \,\text {GeV} , \quad & \Delta p_T > 0 .\end{array}\right. } \end{aligned}$$

The perturbative expansion for the cross section in this case depends only quadratically on the *Z*-boson transverse momentum, and it is interesting to study whether the unphysical behavior as $\Delta p_T\rightarrow 0$ improves in this case. We show the predictions for these product cuts using NNLOJET in Fig. 8. A comparison of the relative difference between the regular cuts and the product cuts at NLO and NNLO is shown in Fig. 9. While the unphysical dependence on $\Delta p_T$ is lessened by switching to product cuts it is still clearly visible on the plots. We note that both the fixed-order and all-orders structure of the cross section with product cuts defined in Eq. (12) are analogous to those discussed in the previous subsections, including the possibility of a mismatch between linear slopes due to the way $\Delta p_T$ is defined here.

### Rapidity distributions

**Fig. 10:**
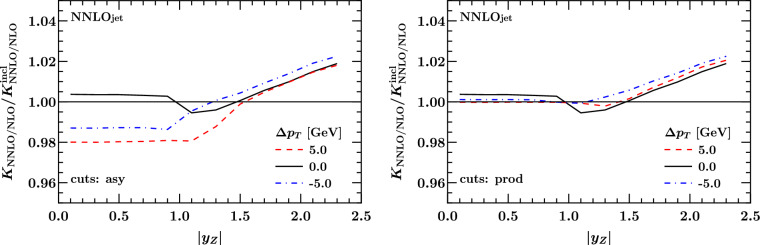
Deviation of the rapidity distribution for $pp \rightarrow Z/\gamma ^*+X \rightarrow l^+l^- + X$ production at $\sqrt{s}=7$ TeV as a function of $\Delta p_T$ relative to the results for $\Delta p_T=0$ at NNLO in QCD computed with NNLOJET. Shown are selected $\Delta p_T$ values (indicated by color) using the definition in Eq. (3) for linear asymmetric cuts (left plot) and the one of Eq. (12) for product cuts (right plot). Solid lines denote negative values of $\Delta p_T$ as indicated in the plots, dotted lines of the same color display the corresponding $\Delta p_T$ values with positive sign

**Fig. 11:**
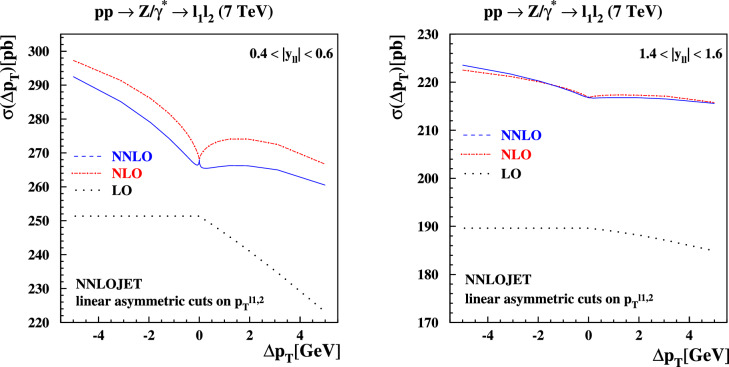
The cross sections for $pp \rightarrow Z/\gamma ^*+X \rightarrow l^+l^- + X$ production at $\sqrt{s}=7$ TeV at LO (dotted), NLO (dashed) and NNLO (solid) in QCD with ABMP16 PDFs computed with NNLOJET as a function of $\Delta p_T \in [-\,5,5]$ GeV defined in Eq. (3) for the linear asymmetric fiducial cuts on the decay leptons in the final state. The rapidity bins $|y_{ll}| \in [0.4,0.6]$ (left plot) and $|y_{ll}| \in [1.4,1.6]$ (right plot) are shown

**Fig. 12:**
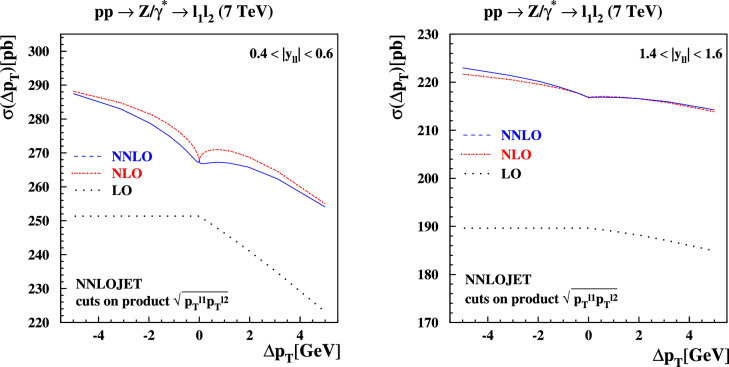
Fiducial NNLO to NLO *K*-factor as a function of $|y_{\ell \ell }|$ for linear asymmetric cuts (left) and product cuts (right) for different values of $\Delta p_T$, normalised to the inclusive NNLO to NLO *K*-factor

It is instructive to revisit the study of the $\Delta p_T$ dependence of the cross section for *Z*-boson production with central leptons, which directly relates to the numerical studies presented in Sect. 2. For selected fixed values of $\Delta p_T$ we display in Fig. 10 the *K*-factor $\sigma _{\textrm{NNLO}}/\sigma _{\textrm{NLO}}$ relative to the inclusive case for asymmetric cuts (left) and product cuts (right) as a function of the *Z*-boson rapidity. In both figures we also display the *K*-factor with symmetric cuts in black.

For the selected values of $\Delta p_T$, we observe a difference at the 2% level across the whole range in rapidity between the asymmetric case and the inclusive case. There is a turning point halfway through the rapidity range considered, with the ratio moving from $\simeq -\,2 \%$ at central rapidity to $\simeq + \, 2 \%$ at $|y_{\ell \ell }| = 2.2$, with an abrupt change in slope starting around $|y_{\ell \ell }| \simeq 1$. The symmetric cuts case displays a similar trend at large $|y_{\ell \ell }|$, while it is closer to the inclusive case at central rapidities. Also in this case, we observe sudden changes in slope at central rapidities, even though the overall deviation with respect to the inclusive case is between [$-$ 0.5%, + 0.5%] for $|y_{\ell \ell }|$ below 1.5.

On the other hand, the product cut curves in Figs. 11, 12 display a smoother behaviour, and they are almost indistinguishable from the inclusive result for $|y_{\ell \ell }|$ below 1.2. Above this value, the *K*-factors start to depart from the inclusive case with a slope similar to the one observed in the asymmetric and the symmetric case, with differences up to $\simeq +2 \%$. For the kinematics under consideration ($pp \rightarrow Z/\gamma ^*+X \rightarrow l^+l^- + X$ production at $\sqrt{s}=7$ TeV with cuts on the decay leptons $p_T^{\ell } \ge 20 \,\text {GeV}$), linear power corrections in $\Delta p_T$ appear for central rapidities, $y_{ll} \lesssim 1.0$, due to the fiducial cuts breaking the azimuthal symmetry in the integral over the lepton decay phase space, as discussed in Refs. [42, 45]. For larger rapidities $y_{ll} \gtrsim 1.5$ the rapidity constraints dominate the integral over the lepton phase space and azimuthal symmetry is restored, resulting in quadratic power corrections in $\Delta p_T$. This leads to only small deviations of all predictions from the case of symmetric cuts. The impact of the cuts on the lepton decay phase space has been illustrated in our previous study [17], including the region around around $y_{ll} \simeq 1.2$, where the transition occurs.^7^

The fact that the product cut *K*-factors display a better behaviour at central rapidities shows that, also at the differential level, product cuts are free of linear power corrections, which are instead responsible for the larger differences observed in the asymmetric case and, to some extent, in the symmetric case. The effect observed are, however, limited to a few percent, and do not display the much larger differences observed in the Higgs case when asymmetric cuts are enforced [51]. Moreover, our analysis is performed at NNLO, while larger shape distortions were observed in the Higgs case at N$^3$LO. A better assessment of the impact of linear power corrections associated with the choice of cuts requires to consider resummation effects at NNLL and beyond, which we will discuss in the next section.

## Resummation

Using resummation techniques, e.g. in the framework of soft-collinear effective theory (SCET), we can quantify the structure of the cross section further. We produce matched predictions of the form

13 $$\begin{aligned} \sigma _\textrm{match} = \sigma _\textrm{FO} + \int _{0}^{q_T^\textrm{off}} \! d q_T \, \left[ \frac{d \sigma _\textrm{res}}{d q_T} - \frac{d \sigma _\textrm{sing}}{d q_T} \right] ,\end{aligned}$$

where $\sigma _\textrm{FO}$ is the fixed-order result, $d \sigma _\textrm{res}/d q_T$ is the all-orders resummed fiducial $q_T$ spectrum, and $d \sigma _\textrm{sing}/d q_T$ is its fixed-order expansion. The latter spectra can be computed by SCETlib using the settings of Ref. [45]. The upper limit $q_T^\textrm{off}$ of the integral is chosen high enough such that all profile scale functions (and thus the resummation) are turned off exactly at $q_T > q_T^\textrm{off}$, and the resummed and singular cross sections cancel exactly beyond that point. In practice we pick $q_T^\textrm{off} = 150 \,\text {GeV}$ such that this criterion is fulfilled even for the highest $m_{\ell \ell } \le 150 \,\text {GeV}$ values that we keep. For our central choice of profile scale transition points, the resummation is actually fully off already for $q_T \ge 0.9 \, m_{\ell \ell }$. For most of the *Z* resonance the cancellation is therefore exact much earlier. It is useful to further define the non-singular cross section

14 $$\begin{aligned} \sigma _\textrm{nons} \equiv \sigma _\textrm{FO} - \int _{0}^{q_T^\textrm{off}} \! d q_T \, \frac{d \sigma _\textrm{sing}}{d q_T} .\end{aligned}$$

The relevant physical settings and cuts on $Q \equiv m_{\ell \ell }$ and $Y \equiv Y_{\ell \ell }$ are given by the input file in Appendix D. Note that we always cut on the lepton pseudorapidities $|\eta _{\ell _1,\ell _2}| \le 2.5$ in addition. The cuts on the lepton transverse momenta are given in Eqs. (3) and (12) for linear asymmetric and product cuts, respectively.

### Structure of the fixed-order cross section and terms captured by resummation

Returning to the general all-orders form of the fixed-order cross section in Eq. (5), it is important to ask which terms are actually being captured and resummed by the singular cross section $d \sigma _\textrm{sing}$ when dividing up the fixed-order cross section as in Eq. (14). In general, we expect that logarithms ($m > 0$ for $\ln ^m(\pm x)$ in Eq. (5)) arise from corresponding logarithmic terms in the expansion of the hadronic structure functions $W_i$ to some (high) power in $q_T/Q$,

15 $$\begin{aligned} 2\pi q_T \, W_i(q^\mu )&= \sum _{n=0}^\infty \alpha _s^n \biggl \{A_{i,n,0}(Q, Y) \, \delta (q_T)\nonumber \\&\quad + \sum _{m > 0}^{2n} A_{i,n,m}(Q,Y) \, \frac{1}{Q} \left[ \frac{\ln ^{m-1} (q_T/Q)}{q_T/Q} \right] _+ \nonumber \\  &\quad + \sum _{k = 0}^\infty (q_T/Q)^k \sum _{m = 0}^{2n} B_{i,n,m}^{(k)}(Q, Y) \ln ^m(q_T/Q) \biggr \}. \end{aligned}$$

Here we work in the notation of Ref. [45], where the inclusive cross section $d \sigma /d^4 q$ is proportional to $W_{-1} + W_0/2$, while for $i \ge 0$ the $W_i \propto A_i \, d \sigma /d^4 q$ are in direct correspondence to the standard angular coefficients $A_i$ up to leptonic prefactors like electroweak charges and electroweak gauge boson propagators. The limit $q_T \ll Q$ is the only relevant source of large logarithms as a function of $q^\mu$, i.e., the momentum transfer between the hadronic and leptonic systems, because other large (threshold) logarithms present in the partonic structure functions are cut off by PDF suppression and the proton–proton kinematics at the hadronic level.

It is easy to verify that for the observable at hand, only the hadronic structure functions $i = -1, 0, 2$ contribute. The leptonic phase space integral vanishes for $i = 3 \dots 7$, while for $i = 1$ it is an odd function of *Y* and thus vanishes when integrated against the even structure function. For these we have

16 $$\begin{aligned} A_{i, n, m}&= 0 , \quad i = 0, 2 , \nonumber \\ B^{(0)}_{i, n, m}&= 0 , \quad i = -1, 0, 2 ,\end{aligned}$$

where the latter relation holds exactly for $i = -1, 0$ and within twist-two collinear factorization for $i = 2$. All the relevant hadronic power corrections to the leading-power factorization predicting the $A_{-1, n, m}$ are thus suppressed by at least two relative powers of $(q_T/Q)^2$ and scale as $(q_T/Q) \ln ^m (q_T/Q)$. Since we expect the leptonic phase space integral to lower the degree of divergence by at least one power, cf. the leading-power and tree-level $\delta (q_T) \sim q_T^{-1}$ being mapped onto the coefficients $c_0 \sim x^0$, $b_{0,0} \, x \sim x^1$, and $a_{0,0} = 0$ in Eq. (5), we conclude that these at most contribute to the terms of ${\mathcal {O}}(x^2)$, while all coefficients $a_{n,m}$ and $b_{n,m}$ for $m > 0$ are predicted in terms of the $A_{-1, n, m}$. These are fully captured by the leading-power (LP) factorized singular cross section,

17 $$\begin{aligned}&\frac{d\sigma _\textrm{sing}}{d Q^2 \, d Y\, d q_T} \propto 2 \pi q_T W_{-1}^\textrm{LP}(q^\mu ) \nonumber \\&\quad = \sum _{n=0}^\infty \alpha _s^n \biggl \{ A_{-1,n,0}(Q, Y) \, \delta (q_T) \nonumber \\&\qquad + \sum _{m > 0}^{2n} A_{-1,n,m}(Q,Y) \, \frac{1}{Q} \biggl [ \frac{\ln ^{m-1} (q_T/Q)}{q_T/Q} \biggr ]_+ \biggl \} ,\end{aligned}$$

where the proportionality again means up to leptonic prefactors. The fiducial singular $q_T$ spectrum entering Eqs. (13) and (14), is then obtained by integrating Eq. (17) over $Q^2$, *Y*, and the leptonic decay phase space, while weighting by $1 + \cos ^2 \theta$ and the fiducial acceptance. In the resummed case, the second line of Eq. (17) is evaluated using the all-orders resummation instead, which we perform as described in Ref. [45].

Importantly, the total offset $c_n$ and the remaining linear slope terms $a_{n,0}$ and $b_{n,0}$ on either side of Eq. (5) are not predicted by this argument because they can arise from rational integrals over contributions from the cross section at any $q_T$. They do not have to arise from integrals over the $1/q_T$ singularity at small $q_T \ll Q$ because they are not transcendental. This is analogous to the case of Ref. [52], where resummation predicted the second derivative of the spectrum of interest, meaning that a constant and a slope term had to be obtained as boundary conditions in a double integral through fixed-order matching. (The discussion in Sect. 3.2 implies that in the case at hand, $a_{n,0}$ and $b_{n,0}$ are not actually connected to each other due to the definition of $\Delta p_T$.) For fiducial Drell–Yan production, the more complicated double integral functional acting on the underlying hadronic structure functions predicted by resummation is instead given by the $q_T$ integral and the constrained leptonic phase-space integral. As in Ref. [52], a natural consequence of this double integral is the presence of “non-singular” terms that feature the same or even higher power counting as the singular terms predicted by factorization, the only distinction being whether they are enhanced by logarithms as $x \rightarrow 0$, i.e., whether their derivative is bounded (non-singular) or logarithmically divergent (singular).

In conclusion, we predict that

18 $$\begin{aligned}&\sigma _\textrm{sing}(x) = \sum _{n=0}^\infty \alpha _s^n \biggl \{ c_n^\textrm{sing} + x \theta (-x) \, a_{n,0}^\textrm{sing} + x \theta (x) \, b_{n,0}^\textrm{sing} \nonumber \\&\qquad + x \theta (-x) \sum _{m = 1}^{2n} a_{n,m} \ln ^m (-x) + x \theta (x) \sum _{m = 1}^{2n} b_{n,m} \ln ^m x + {\mathcal {O}}(x^2) \biggr \} , \nonumber \\&\quad \sigma _\textrm{nons}(x) = \sum _{n=0}^\infty \alpha _s^n \biggl \{ c_n^\textrm{nons} + x \theta (-x) \, a_{n,0}^\textrm{nons} + x \theta (x) \, b_{n,0}^\textrm{nons} + {\mathcal {O}}(x^2) \biggr \} .\end{aligned}$$

Here we have moved the unique prediction of LP factorization ($a_{n,m}$ and $b_{n,m}$ with $m > 0$) to the second line for the singular cross section. In contrast, the precise breakdown of $a_{n,0}$, $b_{n,0}$, and $c_{n}$ between singular and non-singular cross section at fixed order depends on $q_T^\textrm{off}$, the dependence on which cancels between the two terms. Once the singular cross section is resummed, the associated ambiguity is quantified by profile scale variations quoted as $\Delta _\textrm{match}$ in Ref. [45] and below, because they determine down to which values of $q_T$ the resummed singular cross section is equal to its fixed-order counterpart.

In summary, the logarithmic terms $a_{n,m}$ and $b_{n,m}$ for $m \ge 1$ which lead to the unphysical non-monotonic behavior of the fixed-order cross section for $\Delta p_T \ne 0$ appear in the singular cross section. This behavior is therefore cured by resummation. The discontinuity of the cross section derivative for $\Delta p_T = 0$ as encoded in the coefficients $a_{n,0}$, $b_{n,0}$ remains present even after the resummation, in agreement with the general argument of Sect. 3.2. Here the two contributions to the cross section separately obey monotonicity, i.e., $a_{n,0}^\textrm{sing} < 0$, $a_{n,0}^\textrm{nons} < 0$ and $b_{n,0}^\textrm{sing} = b_{n,0}^\textrm{nons} = 0$.

### Numerical results for the non-singular cross section

**Fig. 13:**
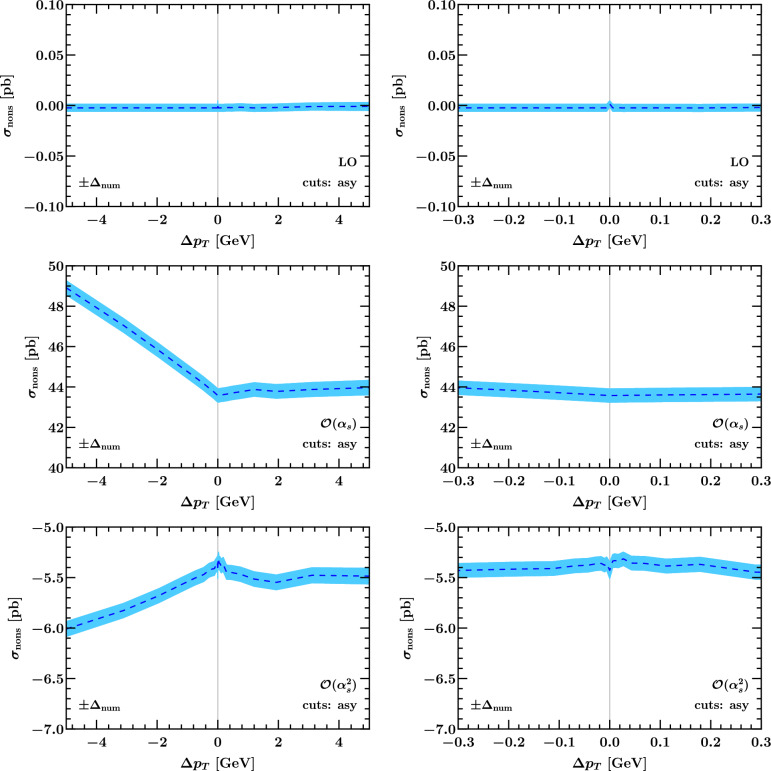
The non-singular cross section with linear asymmetric cuts according to Eq. (3) as a function of $\Delta p_T$ on a wide (left) and zoomed-in scale (right). The blue band indicates the numerical uncertainty, which is predominantly due to the SCETlib integration

**Fig. 14:**
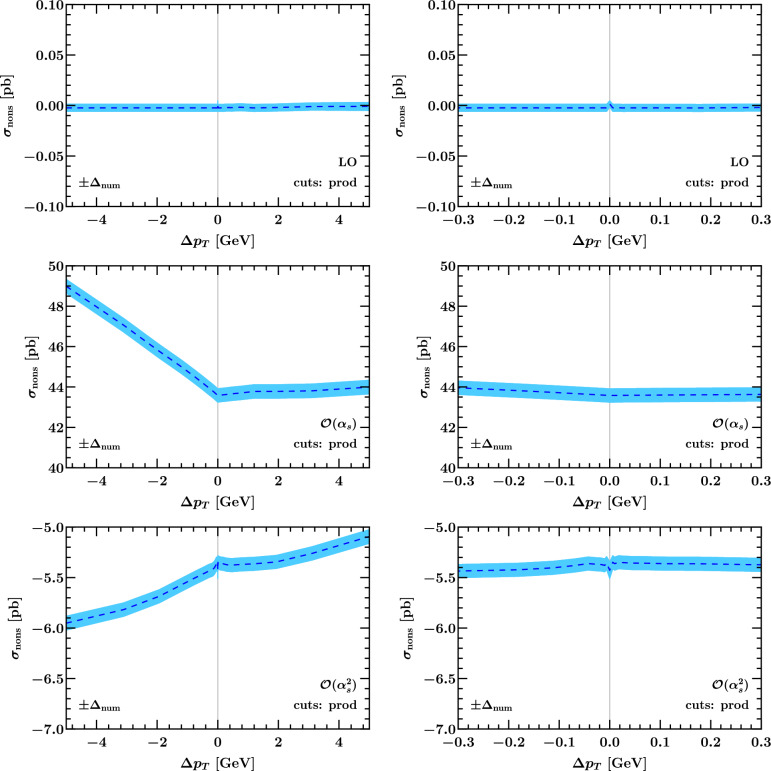
Same as Fig. 13 with product cuts according to Eq. (12)

In Fig. 13 we show the fixed-order perturbative coefficients of the non-singular cross section including the respective factors of $\alpha _s^n$ in the case of the linear asymmetric cuts in Eq. (3). Within the numerical uncertainty, which is indicated by the blue band and driven by the SCETlib beam function interpolation uncertainty, these are all compatible with an asymptotic behavior of two straight lines approaching a constant as $\Delta p_T \rightarrow 0$ with different slopes. The LO non-singular cross section is compatible with zero within a relative accuracy of $10^{-5}$, validating the SCETlib physics inputs against those used at fixed order (in NNLOJET here). We stress that the nonsingular cross section in Eq. (14) is independent of the resummation formalism used. This is because the singular spectrum, whose integral is subtracted from the total fixed-order cross section in Eq. (14), is specified by evaluating the underlying hadronic structure functions in *fixed-order perturbation theory* and expanding them to leading power in $q_T/Q$, while keeping the exact dependence on $q_T$ in the leptonic phase space integral. The choice to evaluate the structure functions in the Collins–Soper frame and the choice to expand in 1/*Q* (instead of the inverse of the transverse mass) amount to quadratic power corrections. Due to their lower degree of divergence at the level of the $q_T$ spectrum, these cannot induce logarithmic dependence on $\Delta p_T$, and thus these choices (like the choice of $q_T^\textrm{off}$) only affect the constant terms and linear slopes in Eq. (14). For the case of product cuts defined in Eq. (12) the non-singular cross sections at LO, NLO and NNLO are plotted in Fig. 14. The only notable feature compared to Fig. 13 is a faster (possibly quadratic) rise of the $\alpha _s^2$ coefficient towards large positive values of $\Delta p_T$, but this is at the scale of a few tenths of a picobarn.

### Matched predictions

**Fig. 15:**
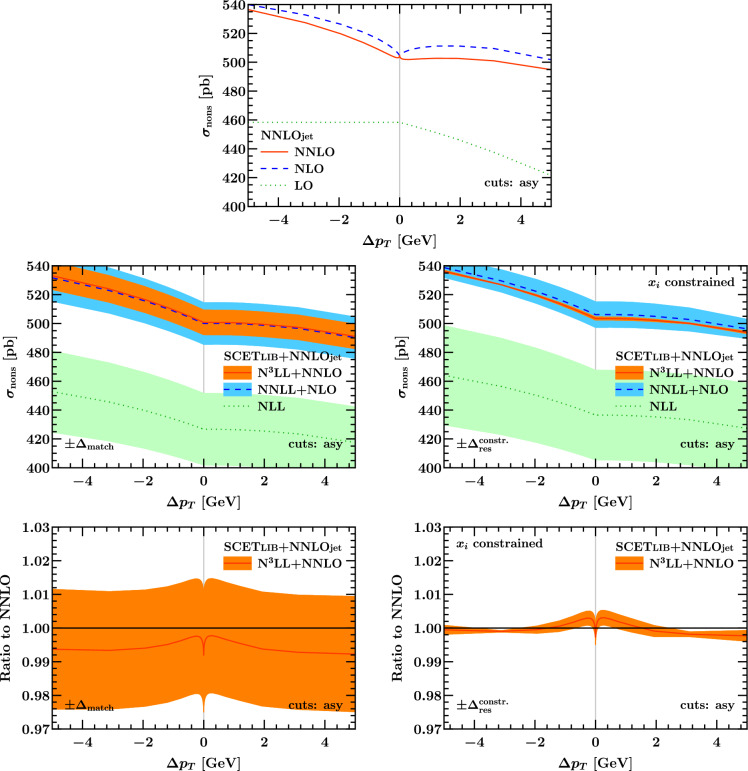
Results for the total fiducial cross section with linear asymmetric cuts according to Eq. (3) as a function of $\Delta p_T$ on a wide (left) and zoomed-in scale (right). The bottom row shows the ratio to the NNLO prediction

**Fig. 16:**
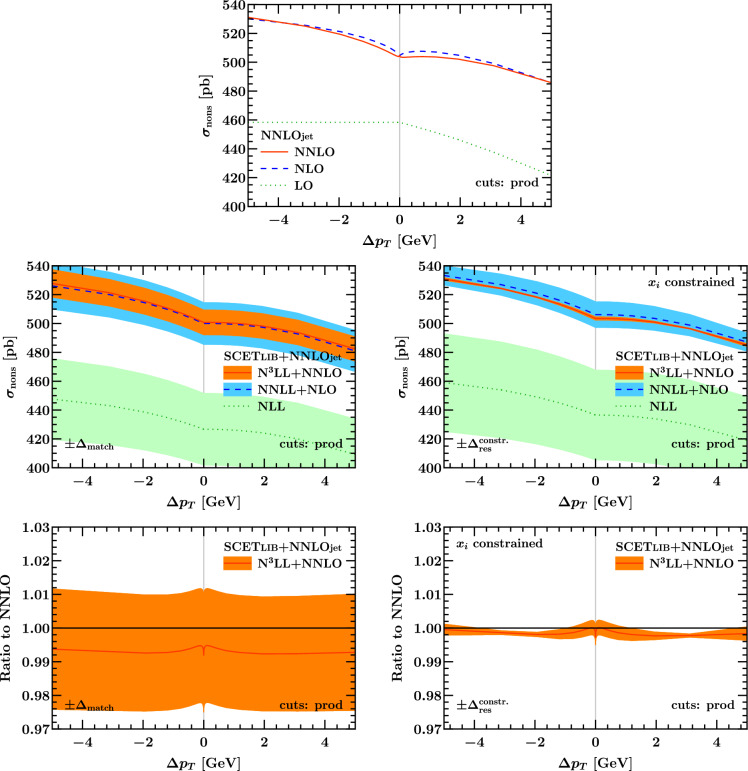
Same as Fig. 15 with product cuts according to Eq. (12)

In Fig. 15 we show predictions for the fiducial cross section at different fixed (top) and resummed and matched orders (bottom). It is clear that the fixed-order predictions are neither monotonic, nor do they have a vanishing derivative as $\Delta p_T \rightarrow 0^+$. Both of these physical properties are restored by the resummation. The resummed and matched cross section still feature a discontinuous derivative at $\Delta p_T = 0$, but this is in fact a physical feature and a consequence of the definition of $\Delta p_T$ as discussed previously. The resummed and matched predictions are also monotonically decreasing and have vanishing derivative as $\Delta p_T \rightarrow 0^+$.

The resummation is under good theoretical control, as evidenced by the matching uncertainty $\Delta _\textrm{match}$, which we plot together with the matched predictions in the left column of Fig. 15. Here $\Delta _\textrm{match}$ is estimated by profile scale variations using the formalism and default settings introduced in Ref. [45], and features excellent perturbative convergence and coverage. For later reference, we briefly describe the salient features of this default matching formalism. Reference [45] employs a hybrid profile scale technique [53], where up to a value of $q_T = x_1 Q$ the resummation is fully turned on. In this first region, the spectrum is obtained from a numerical Fourier transform of the resummed $b_T$-space cross section. The beam and soft functions are evaluated at the so-called canonical boundary values for their virtuality and rapidity scales,

$$\begin{aligned} \mu _S(b_T) = \mu _B(b_T) = \nu _S(b_T) = \frac{b_0}{b_T} , \qquad \nu _B = Q ,\end{aligned}$$

where $b_0 = 2e^{-\gamma _E}$ and $\gamma _E$ is the Euler–Mascheroni constant. An identical result for the resummed $b_T$-space cross section is obtained when choosing canonical $\mu$ and Collins–Soper scales $\zeta$ in TMD factorization. For $q_T \ge x_1 Q$, the resummation is slowly turned off by promoting the scales in Eq. (19) to be functions of $q_T$ that continuously and monotonically approach the fixed-order value $\mu _R = \mu _F = Q$ for all values of $b_T$ as $q_T \rightarrow Q$. Specifically, one picks another point $q_T = x_3 Q > x_1 Q$ beyond which all scales are equal to *Q*, the resummation is fully off, and the fixed-order result for the spectrum is recovered exactly. A third intermediate point $q_T = x_2 Q$ with $x_1< x_2 < x_3$ relates to the precise functional form of the transition and governs how quickly it departs from the canonical resummation region. The default settings for these parameters from Ref. [45], which were determined quantitatively by comparing the size of leading-power singular and the nonsingular terms in the $q_T$ spectrum, are given by

19 $$\begin{aligned} (x_1, x_2, x_3) = (0.3, 0.6, 0.9) .\end{aligned}$$

The matching uncertainty $\Delta _\textrm{match}$ is then estimated by varying them as

20 $$\begin{aligned}&(x_1, x_2, x_3) \in V_\textrm{match} = \{ (0.4, 0.75, 1.1),\, \nonumber \\&(0.2, 0.45, 0.7),\, (0.4, 0.55, 0.7),\, (0.2, 0.65, 1.1)\} ,\end{aligned}$$

i.e., one either shifts the transition region up, shifts it down, condenses it, or stretches it out. Notably, the relative size of singular and nonsingular contributions from which these default choices were determined was assessed at the level of the $q_T$ *spectrum* in Ref. [45] in order to obtain an optimal prediction for its shape. By contrast, neither the central value of the $x_i$ nor their variations were tuned to exactly preserve the fixed-order prediction for the *integral* of the inclusive $q_T$ spectrum, i.e., the total inclusive cross section. As an important cross check, we have therefore explicitly verified that the above default resummation and matching setup nevertheless leads to a net resummation effect compatible with zero when applied to the *inclusive* cross section within the ATLAS $Q^2$ and *Y* bins. Explicitly, we find

21 $$\begin{aligned} \sigma ^\text {incl}_\text {res,NLL} - \sigma ^\text {incl}_\text {sing,LO}&= (-12.5 \pm _\textrm{match} 42.7) \, \text {pb} , \nonumber \\ \sigma ^\text {incl}_\text {res,NNLL} - \sigma ^\text {incl}_\text {sing,NLO}&= (-5.6 \pm _\textrm{match} 25.5) \, \text {pb} , \nonumber \\ \sigma ^\text {incl}_{\text {res,N}^3\text {LL}} - \sigma ^\text {incl}_\text {sing,NNLO}&= (-5.2 \pm _\textrm{match} 15.1) \, \text {pb} , \end{aligned}$$

where $\pm _\textrm{match}$ indicates the matching uncertainty $\Delta _\textrm{match}$ at the given order.

As an alternative to the default spectrum-level setup from Ref. [45], one may instead consider profile scales that are chosen such that the matching *exactly* preserves the total inclusive cross section as computed at a given fixed order in perturbation theory. We stress that because the default matching is implemented through profile scales, the nonzero resummation effects in Eq. (21) are by construction also beyond the order at which the fixed-order calculation is truncated. To do so we maintain the original functional form of the transition as given in Ref. [45], but choose the transition points $x_i$ under the constraint that for the central prediction (which uses Eq. (19) in the canonical region $q_T \le x_1 Q$), the integral of the matched cross section up to $q_T = x_3 Q$ exactly recovers the fixed-order value. We further choose to hold $x_1 = 0.3$ fixed for the central prediction such that the size of the canonical region coincides with the default settings. By manually scanning the dependence on the other two parameters, we then find that

22 $$\begin{aligned} (x_1, x_2, x_3)_\mathrm {constr.} = (0.3, 0.9, 1.2) \end{aligned}$$

exactly recovers (for the settings used in this paper, and in particular the PDF set at hand) the NNLO total inclusive cross section from the integral of the N$^3$LL$+$NNLO inclusive $q_T$ spectrum.

It is interesting to ask how an uncertainty estimate based on scale variations can still be obtained for the $q_T$ spectrum in this case, as well as for derived quantities like fiducial cross sections that feature sensitivity to resummation effects, since the matching uncertainty has largely become trivial by imposing the above constraint. In particular, these additional variations should probe possible changes of the shape of the spectrum in the canonical region itself, which can also serve as a proxy for the difference between different resummation formalisms. To test this we adapt another, separate component of the total uncertainty estimate for the $q_T$ spectrum from Ref. [45], specifically, the so-called resummation uncertainty $\Delta _\textrm{res}$. Conventionally, $\Delta _\textrm{res}$ is estimated by performing a large set of variations where the four canonical scales in Eq. (19) are varied independently or jointly by factors of 2 around their central values [54]. Taking an envelope of these variations then provides one of the most detailed estimates of the residual uncertainty on the spectrum in resummed perturbation theory available within the scale variation paradigm. While these variations are smoothly turned off as $q_T$ increases such that the fixed-order prediction in the tail of the spectrum is unaffected by them, they again do not necessarily preserve the total integral. While they probe variations from the canonical Sudakov shape in depth, they are not necessarily a pure *shape* variation of the spectrum. To complete our uncertainty estimate in the case where the $x_i$ are constrained, we also subject the variations entering $\Delta _\textrm{res}$ to the integral constraint by adjusting the $x_i$ for each variation. Restricting to a subset of representative variations to keep the complexity of the problem manageable, we find

23 $$\begin{aligned} V_\textrm{res}^{\mathrm{constr.}}&= \bigl \{ (\mu _B^\textrm{down}, \mu _S^\textrm{down}, 0.55, 1.0, 1.2), \nonumber \\&\qquad (\mu _B^\textrm{up}, \mu _S^\textrm{up}, 0.25, 0.65, 1.2), \, \nonumber \\&\qquad (\nu _B^\textrm{down}, 0.35, 0.9, 1.2), \, (\nu _B^\textrm{up}, 0.4, 0.8, 1.2) \bigr \} ,\end{aligned}$$

where $\mu _X^{\textrm{up,down}}$ indicates a soft or beam function scale variation as described in Ref. [45], and the three values quoted are the respective $x_i$. Note that in this case, we have held $x_3$ fixed at the central value determined earlier to ensure that all variations in $V_\textrm{res}^{\mathrm{constr.}}$ collapse onto the fixed-order spectrum at the same point, and instead compensated the changes by mainly varying $x_1$, i.e., the size of the purely canonical region.

The results of the above exercise are shown in the right column of Fig. 15. Compared to the left column, the central values of the matched predictions are shifted up, largely compensating – now at the fiducial level – the small offsets we reported in Eq. (21). Note that since we are mainly interested in the behavior at the highest order in perturbation theory, we have used the same constrained variations from above at all three orders shown in Fig. 15 for expediency. This is the reason for the much larger uncertainty bands at lower orders. Nevertheless, as can be seen from the bottom right panel, we still find a small net resummation effect on the central value at N$^3$LL$+$NNLO that is mainly compatible with zero, but just outside the refined uncertainty estimate for some values of $\Delta p_T$. While a complete study of this effect at the next higher order is beyond the scope of this paper, we anticipate that the effect will become more significant at higher orders since the estimate for $\Delta _\textrm{res}^\mathrm {constr.}$ will be reduced as the residual scale dependence decreases in the resummed cross section, while the baseline fixed-order prediction picks up two additional logarithms of $\Delta p_T/Q$ at each order according to Eq. (18).

Another uncertainty intrinsic to the resummed prediction is given by the impact of non-perturbative transverse momentum dependent (TMD) physics and the Landau pole in the inverse Fourier transform, which is estimated by variations of a cutoff parameter $\Lambda _\textrm{fr}$ defined in Ref. [45]. We find that the resulting $\Delta _\textrm{NP}$ is not even resolved at the current relative numerical uncertainty, which is ${{{\mathcal {O}}}}(10^{-4})$. This is expected because for typical $q_T \sim 25 \,\text {GeV}$ in the baseline symmetric cuts (which dominate the total fiducial cross section here), the effect of non-perturbative TMD physics is suppressed by $(\Lambda _\textrm{QCD}/q_T)^2 \sim (500 \,\text {MeV} / 25 \,\text {GeV})^2 \sim 4 \times 10^{-4}$.

Figure 16 shows our results for the matched cross sections for the case of the product cuts defined in Eq. (12), also compared to the NNLOJET fixed-order results. They are again almost identical to their counterparts for linear asymmetric cuts in Fig. 15. A few comments on the product cuts are in order. Reference [22] focused on constructing $1 \rightarrow 2$ fiducial cuts that would be free of ${\mathcal {O}}(q_T)$ linear power corrections at the level of the differential spectrum as $q_T \rightarrow 0$, which were found to reduce ambiguities in the perturbative series for the case of Higgs production with $\Delta p_T \sim 10$ GeV. Furthermore, the absence of linear power corrections in the acceptance is beneficial for mitigating bias in fixed-order subtraction methods. However, the sensitivity of the total fiducial cross section to resummation effects remains present through terms of the form $x \ln ^n x$ for $x = \Delta p_T/M_Z \ll 1$. This is evident from our numerical results in Fig. 8, where the unphysical behavior for $\Delta p_T \rightarrow 0$ of the fixed-order cross section persists also for product cuts. We note that this observation does not straightforwardly follow from the definition of product cuts. Although they trivially coincide with the symmetric cuts for $\Delta p_T \rightarrow 0$, they might have displayed a different asymptotic behavior approaching that limit. In the language of Ref. [45], there are two classes of fiducial power corrections, either “linear” or “leptonic”, which do not have to coincide. “Leptonic” power corrections in $q_T/\Delta p_T = x q_T/M_Z$ are in general present and lead to sensitivity to low values of $q_T$ at any power in $q_T/\Delta p_T$. Ultimately, the only way to cure the pathological behavior in the $\Delta p_T = 0$ limit is to resort to resummation, due to the presence of $x \ln x$ power corrections for $q_T/M_Z \lesssim x$, as already observed in [21].

### Rapidity distributions

**Fig. 17:**
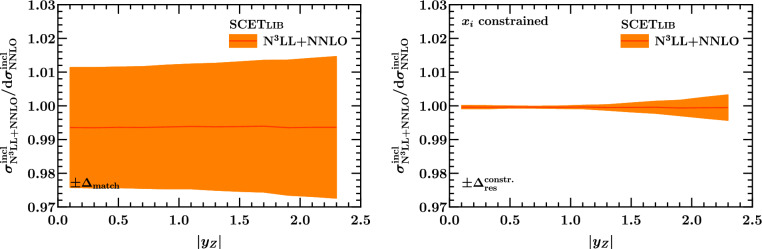
Net resummation effect on the inclusive rapidity spectrum, using either the default matching setup of Ref. [45] with its associated matching uncertainty $\Delta _\textrm{match}$ (left) or the integral-preserving matching setup described in Sect. 4.3 featuring a constrained resummation uncertainty $\Delta _\textrm{res}^\mathrm {constr.}$ (right)

We are now in a position to assess the size of resummation effects on the fiducial rapidity distributions studied at fixed order in Sect. 3.4. As a baseline, we first assess the impact of the resummation on the inclusive rapidity spectra using the two approaches described in Sect. 4.3 to perform the matching. The results at N$^3$LL$+$NNLO are shown in Fig. 17. For the default matching setup of Ref. [45], results from which are shown in the left panel, we find that the integral is compatible with the fixed-order value within the matching uncertainty for all rapidities, providing us with a rapidity-differential version of the check in Eq. (21). The results using the constrained profile scales and the constrained resummation uncertainty introduced in Sect. 4.3 are shown in the right panel. Note that the constraint on the transition points $x_i$ was applied at the level of the total inclusive cross section in the ATLAS *Q* and central *Y* bin, and not point by point in rapidity. This leads to the finite value of $\Delta _\textrm{res}^\mathrm {constr.}/\sigma _\textrm{LO} \approx 0.4 \%$ at $1.5 \le |y_Z| < 2.5$, where the effect of the beam function $\mu _B$ variation changes due to the changing behavior of the underlying PDFs. While it would in principle be possible to apply the constraint point by point in $y_Z$ (and *Q*), developing the required automated numerical framework, e.g. along the lines of Ref. [55], is beyond the scope of this exercise here. Here we simply point out that for all $y_Z$, the integral of the constrained matched prediction is indeed compatible with the fixed-order result within the residual $\Delta _\textrm{res}^\mathrm {constr.}$.

**Fig. 18:**
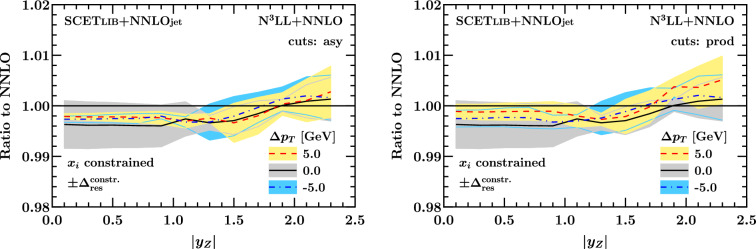
Net resummation effect on the fiducial *Z* rapidity spectrum using asymmetric cuts (left) or product cuts (right) as a function of $|y_Z|$ for representative values of $\Delta p_T$. For clarity, we only show results for the constrained matching setup. (The results from the default matching setup are not very instructive at this resolution.)

Turning to the fiducial *Z* rapidity spectrum, we first show the effect of resummation for representative values of $\Delta p_T$ as a function of $y_Z$ in Fig. 18. Our results for $\Delta p_T = 0$ confirm the observations of Ref. [56]: the resummation has a small negative net effect of $-\,0.4 \%$ at central rapidities, then starts to rise at the transition point $|y_Z| \sim 1.2$ identified in Sect. 3.4, and eventually contributes a small positive net effect of $+ 0.2\%$ at $|y_Z| = 2.5$. (Note that in order to read this behavior off from the respective figure in Ref. [56], one must compare the resummation to the *unbiased* fixed-order result using a recoil prescription.) The fiducial rapidity spectra for $\Delta p_T = \pm 5\,\textrm{GeV}$ feature a very similar trend. We note, however, that the resummation effect at this order is still compatible with zero for most values of $\Delta p_T$ and $y_Z$ within $\Delta _\textrm{res}^\mathrm {constr.}$. To further corroborate these findings, we also compute the net resummation effect on the fiducial $y_Z$ spectrum for different $\Delta p_T$ using the RadISH resummation code [11] and the settings of Ref. [13]. We find excellent agreement with Fig. 18. This result complements and extends the fixed-order analysis performed in Sect. 3.4, showing that when NNLO is used as the baseline, the resummation of linear power corrections has a small effect. This suggests that beyond NNLO, the choice of cuts has an effect at the subpercent level, independently of the presence or absence of linear power corrections, and is likely smaller than the N$^3$LO correction, which has been found to be around $-\,2\%$ for different choices of cuts in [13].

**Fig. 19:**
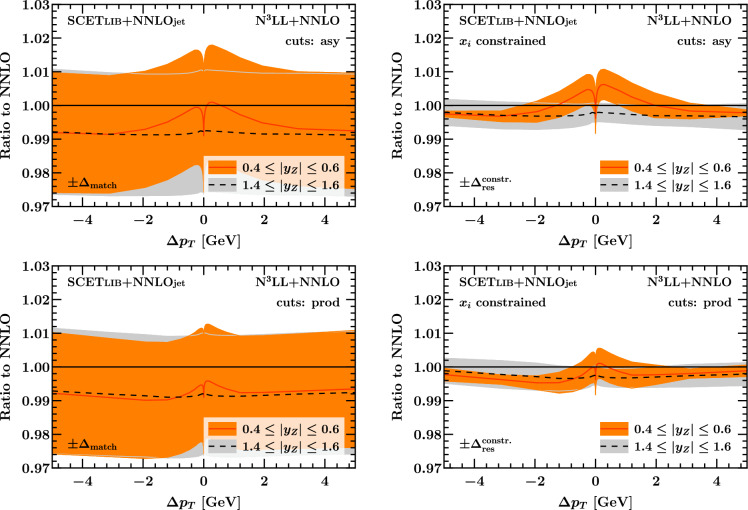
Net resummation effect on the fiducial *Z* rapidity spectrum using asymmetric cuts (top) or product cuts (bottom) as a function of $\Delta p_T$ for two representative bins in $|y_Z|$. We show results using the default matching setup (left) and the integral-constrained matching setup (right)

In Fig. 19 we show the effect of the resummation on the fiducial rapidity spectrum in the two representative bins $0.4 \le |y_Z| \le 0.6$ and $1.4 \le |y_Z| \le 1.6$ considered earlier in Sect. 3.4. In this case we also show results from the default matching setup for comparison. We find that in the more forward bin (gray), which lies past the transition point at $|y_Z| \approx 1.2$, the asymmetric and product cuts both show a very similar behavior as a function of $\Delta p_T$, with a net resummation effect of $-\,0.2\%$ (assuming the constrained matching setup) that is compatible with zero within $\Delta _\textrm{res}^\mathrm {constr.}$. This is expected since for these values of $|y_Z|$, linear power corrections are absent in either case because the lepton pseudorapidity cuts dominate. The picture is different for the central rapidity bin (orange), where the net resummation effect closely resembles that of the total cross section shown earlier in Figs. 15 and 16. In this case, albeit being tiny, the net resummation effect of $\approx -\,0.3 \%$ in the central $|y_Z|$ bin is not compatible with zero within uncertainties for both sets of cuts in the region of $\Delta p_T$ between $-\,5$ and $-\,2 \, \textrm{GeV}$.

## Experimental resolution

From the previous discussions and numerical results we see that fixed-order codes exhibit an unphysical behavior for symmetric cuts that is remedied by resummation. These findings need to be put in perspective of the resolution in the collider experiments, in particular at the LHC. To quantify this we refer to Fig. 15 (bottom row), where the ratios of the resummed result to the NNLO predictions are shown for the observable under study, i.e. the cross sections for $pp \rightarrow Z/\gamma ^*+X \rightarrow \ell ^+\ell ^- + X$ production at $\sqrt{s}=7$ TeV integrated over rapidity in the range $y_{\ell \ell } \le 2.5$, and with fiducial cuts on the decay leptons staggered by $\Delta p_T$. For large $\Delta p_T$ the difference between the resummed and fixed-order asymptotes to approximately 0.4%. At values for $\Delta p_T$ of a few hundred MeV the difference is 0.2%. Given the good perturbative convergence, we note that this effect is small upon inclusion of the NNLO fixed-order corrections and that the differences between fixed-order and resummed predictions are significantly smaller than the residual theoretical uncertainties from scale variations, cf. Fig. 15 (center row). The observed differences are also smaller than other theoretical uncertainties such as those coming from PDFs, see, e.g. Ref. [3].

On the experimental side, the sensitivity to the QCD effects due to symmetric cuts is related to the lepton energy and momentum calibration, and the detector performance. The ATLAS collaboration reports an experimental resolution on the lepton transverse momenta in the range around 20–30 GeV of a few percent (3–4% for electrons [57] and 2 to 3% for muons [58]) together with a systematic uncertainty in the lepton energy scale calibration in the range between 0.03 to 0.2% around the *Z*-boson peak (electrons with transverse momentum close to 45 GeV) [57, 58], cf. [59] for related studies of the CMS collaboration.

In comparison to the current overall experimental precision reported for the inclusive Drell–Yan cross section, for which systematic uncertainties approaching 2% are reported [60, 61], the differences from symmetric cuts treated at fixed-order in perturbative QCD or resummed to all orders are also small. For inclusive cross sections the experimental precision is limited by the uncertainties in the luminosity determination, which are typically around 1.5–2.5%. The latest ATLAS luminosity calibration [62] has been able to achieve 1% accuracy on the luminosity in Run 3, which is expected to be a lower limit on the luminosity uncertainty for Run 3 and the high-luminosity runs. Our NNLO analysis indicates that the all-order resummation of the logarithms in the fiducial transverse momentum spectrum and the unphysical behavior of fixed-order perturbation theory are unlikely to have an impact on experimental studies of inclusive Drell–Yan cross sections. The findings of this study should eventually be corroborated by a N$^3$LO analysis, although the impact of resummation seems to remain moderate at higher orders (see e.g. Ref. [13]).

For differential distributions or normalized cross sections the situation is different, because the effect of the luminosity uncertainty cancels or leads to a coherent shift, and the precision on the shape or ratio of distributions can be much higher. In fact, already for the ATLAS measurement [23] considered in the benchmark study in Sect. 2 a precision on the *Z*-boson rapidity distribution in the central region at the level of a few per-mille is reported, excluding the luminosity uncertainty. Thus, for shapes or ratios of differential distributions $q_T$-resummation can have an impact on the theoretical predictions, cf. [56]. As the dominant uncertainties from the lepton calibrations (*Z*-boson) and from backgrounds and recoil calibration (*W*-boson) are reduced in future measurements of differential distributions for Drell–Yan processes, the differences between fixed-order perturbative QCD and resummed predictions for symmetric fiducial cuts should be revisited.

## Conclusions and discussion

Fixed-order QCD perturbation theory for the Drell–Yan process suffers from an instability for symmetric cuts on the transverse momenta of the final-state leptons. This pathology originates from a logarithmically enhanced power correction present for small lepton-pair transverse momentum. It leads to unphysical non-monotonic behavior for the cross section as the difference $\Delta p_T$ between the cuts on the lepton and anti-lepton is varied. Available public codes that compute the fixed-order NNLO QCD corrections to Drell–Yan treat this instability in different ways due to the underlying subtraction scheme in the codes, potentially leading to different theoretical predictions for the cross section and hindering the analysis of experimental data.

In this paper we have performed a detailed analysis of this issue, involving both the careful study of available fixed-order codes, the resummation of QCD perturbation theory to solve this issue, and the consideration of product cuts that have been suggested as a resolution of this issue. We have considered the following codes that compute the NNLO QCD corrections to the Drell–Yan process:

- DYTURBO (version 1.2), based on the non-local $q_T$ subtraction scheme;
- FEWZ (version 3.1), based on the local sector decomposition subtraction scheme;
- MATRIX (version 2.1), based on the non-local $q_T$ subtraction scheme;
- NNLOJET, based on the local antenna subtraction scheme.

All codes, whether based on local or non-local subtraction schemes, give consistent results for the case of symmetric cuts. This is a non-trivial result since these techniques treat the $p_T=0$ region very differently. Obtaining the correct fixed-order result with non-local schemes requires a careful treatment of power corrections for small transverse momentum. The agreement between these codes is a testament to the community effort exerted for a proper theoretical treatment of this process, with DYTURBO, FEWZ and MATRIX being readily available to the public.

**Table 1:** Cross sections at LO, NLO and NNLO in QCD in fb for inclusive $pp \rightarrow W^+ + X \rightarrow l^+ \nu + X$ production at $\sqrt{s}=7$ TeV, subject to the fiducial cuts applied by measured by the ATLAS experiment [23]. See also Sect. 2 for the settings. Numbers in round brackets indicate the statistical uncertainty from the Monte Carlo evaluation on the last digits

min $|y_l|$	cntr $|y_l|$	max $|y_l|$	$\sigma _{\textrm{LO}}$ [fb]	$\sigma _{\textrm{NLO}}$ [fb]	$\sigma _{\textrm{NNLO}}$ [fb]
0	0.105	0.21	1,144,924 (22)	1,131,302 (49)	1,132,372 (218)
0.21	0.315	0.42	1,147,528 (23)	1,133,998 (58)	1,135,481 (300)
0.42	0.525	0.63	1,152,891 (23)	1,139,771 (59)	1,141,365 (307)
0.63	0.735	0.84	1,160,339 (23)	1,147,812 (59)	1,149,361 (327)
0.84	0.945	1.05	1,169,448 (23)	1,157,995 (60)	1,159,151 (331)
1.05	1.21	1.37	1,181,246 (19)	1,172,429 (44)	1,173,206 (228)
1.37	1.445	1.52	1,189,295 (29)	1,184,195 (80)	1,183,306 (473)
1.52	1.63	1.74	1,190,632 (24)	1,190,271 (59)	1,186,759 (319)
1.74	1.845	1.95	1,182,394 (25)	1,189,693 (61)	1,183,688 (353)
1.95	2.065	2.18	1,155,801 (24)	1,173,921 (57)	1,163,509 (326)
2.18	2.34	2.5	1,082,093 (22)	1,117,404 (46)	1,102,564 (225)

**Table 2:** Same as Table 1 for inclusive $pp \rightarrow W^- + X \rightarrow l^- \nu + X$ production at $\sqrt{s}=7$ TeV

min $|y_l|$	cntr $|y_l|$	max $|y_l|$	$\sigma _{\textrm{LO}}$ [fb]	$\sigma _{\textrm{NLO}}$ [fb]	$\sigma _{\textrm{NNLO}}$ [fb]
0	0.105	0.21	848,830 (14)	862,143 (28)	855,642 (122)
0.21	0.315	0.42	844,251 (14)	857,976 (32)	851,304 (174)
0.42	0.525	0.63	835,145 (14)	849,539 (32)	843,000 (173)
0.63	0.735	0.84	821,844 (14)	837,206 (32)	830,928 (165)
0.84	0.945	1.05	804,614 (14)	821,162 (32)	815,662 (161)
1.05	1.21	1.37	777,824 (11)	795,869 (23)	790,738 (112)
1.37	1.445	1.52	750,484 (16)	769,521 (43)	764,996 (221)
1.52	1.63	1.74	726,685 (14)	745,954 (32)	741,848 (162)
1.74	1.845	1.95	697,056 (14)	716,004 (32)	712,404 (173)
1.95	2.065	2.18	664,666 (14)	682,198 (32)	678,928 (156)
2.18	2.34	2.5	620,643 (13)	634,655 (27)	631,338 (118)

**Table 3:** Same as Table 1 for central inclusive $pp \rightarrow Z + X \rightarrow l^+l^- + X$ production at $\sqrt{s}=7$ TeV

min $|y_{ll}|$	cntr $|y_{ll}|$	max $|y_{ll}|$	$\sigma _{\textrm{LO}}$ [fb]	$\sigma _{\textrm{NLO}}$ [fb]	$\sigma _{\textrm{NNLO}}$ [fb]
0	0.1	0.2	242,501 (4)	260,269 (6)	260,299 (11)
0.2	0.3	0.4	241,885 (4)	259,761 (6)	259,821 (12)
0.4	0.5	0.6	240,596 (4)	258,581 (5)	258,698 (11)
0.6	0.7	0.8	238,771 (4)	256,990 (6)	257,210 (11)
0.8	0.9	1.0	236,249 (4)	254,824 (6)	255,082 (11)
1.0	1.1	1.2	227,905 (4)	251,037 (6)	249,373 (11)
1.2	1.3	1.4	207,158 (4)	234,939 (5)	233,888 (12)
1.4	1.5	1.6	179,949 (4)	208,041 (5)	208,362 (13)
1.6	1.7	1.8	147,438 (3)	173,199 (5)	174,566 (13)
1.8	1.9	2.0	110,916 (3)	132,203 (5)	134005 (14)
2.0	2.1	2.2	72,370 (3)	87,305 (5)	88,905 (13)
2.2	2.3	2.4	34,732 (3)	42,185 (4)	43,064 (11)

**Table 4:** Same as Table 1 for forward inclusive $pp \rightarrow Z + X \rightarrow l^+l^- + X$ production at $\sqrt{s}=7$ TeV

min $|y_{ll}|$	cntr $|y_{ll}|$	max $|y_{ll}|$	$\sigma _{\textrm{LO}}$ [fb]	$\sigma _{\textrm{NLO}}$ [fb]	$\sigma _{\textrm{NNLO}}$ [fb]
1.2	1.3	1.4	22,646 (1)	13,899 (3)	15,537 (15)
1.4	1.5	1.6	45,488 (2)	36,627 (4)	36,949 (16)
1.6	1.7	1.8	72,941 (2)	66,461 (4)	65,760 (15)
1.8	1.9	2.0	103,456 (2)	101,449 (4)	100,231 (15)
2.0	2.1	2.2	134,086 (2)	13,815 (4)	13,692 (15)
2.2	2.3	2.4	161,961 (2)	17,286 (4)	17,205 (13)
2.4	2.6	2.8	157,264 (2)	17,023 (3)	16,929 (9)
2.8	3.0	3.2	77,063 (1)	80,140 (2)	78,526 (8)
3.2	3.4	3.6	20,432 (1)	20,227 (1)	19,374 (6)

**Table 5:** Cross sections at LO, NLO and NNLO in QCD in fb for inclusive $p{{\bar{p}}} \rightarrow W^+ + X \rightarrow l^+ \nu + X$ production at $\sqrt{s}=1.96$ TeV, subject to the fiducial cuts applied by the DØ experiment [24]. See also Sect. 2 for the settings. Numbers in round brackets indicate the statistical uncertainty from the Monte Carlo evaluation on the last digits

min $y_l$	cntr $y_l$	max $y_l$	$\sigma _{\textrm{LO}}$ [fb]	$\sigma _{\textrm{NLO}}$ [fb]	$\sigma _{\textrm{NNLO}}$ [fb]
− 3.2	− 2.95	− 2.7	20,546 (1)	20,997 (3)	20,789 (7)
− 2.7	− 2.55	− 2.4	47,756 (1)	50,253 (5)	50,219 (17)
− 2.4	− 2.3	− 2.2	68,277 (3)	72,825 (8)	73,124 (29)
− 2.2	− 2.1	− 2.0	84,664 (3)	91,041 (9)	91,660 (30)
− 2.0	− 1.9	− 1.8	100,108 (3)	108,305 (9)	109,294 (33)
− 1.8	− 1.7	− 1.6	114,218 (3)	124,151 (10)	125,558 (33)
− 1.6	− 1.4	− 1.2	132,796 (2)	144,935 (6)	146,797 (18)
− 1.2	− 1.1	− 1.0	149,451 (3)	163,439 (11)	165,639 (35)
− 1.0	− 0.9	− 0.8	159,754 (4)	174,717 (11)	177,081 (36)
− 0.8	− 0.7	− 0.6	169,663 (4)	185,463 (12)	187,936 (38)
− 0.6	− 0.5	− 0.4	179,264 (4)	195,794 (12)	198,292 (38)
− 0.4	− 0.3	− 0.2	188,493 (4)	205,663 (12)	208,161 (40)
− 0.2	− 0.1	0.0	197,178 (4)	215,042 (13)	217,563 (42)
0.0	0.1	0.2	204,955 (5)	223,597 (13)	225,922 (44)
0.2	0.3	0.4	211,345 (5)	230,892 (13)	233,051 (44)
0.4	0.5	0.6	215,610 (5)	236,284 (14)	238,207 (47)
0.6	0.7	0.8	216,713 (5)	238,739 (14)	240,426 (48)
0.8	0.9	1.0	213,411 (5)	236,982 (14)	238,314 (48)
1.0	1.1	1.2	204,339 (5)	229,334 (14)	230,561 (46)
1.2	1.4	1.6	176,867 (3)	202,614 (7)	203,859 (22)
1.6	1.7	1.8	136,734 (4)	160,146 (11)	161,689 (37)
1.8	1.9	2.0	105,594 (3)	125,021 (9)	126,689 (30)
2.0	2.1	2.2	75,804 (3)	90,298 (8)	91,681 (23)
2.2	2.3	2.4	50,319 (2)	60,105 (5)	61,155 (17)
2.4	2.55	2.7	26,925 (1)	32,183 (3)	32,744 (7)
2.7	2.95	3.2	7572 (0)	9011 (1)	9178 (2)

**Table 6:** Same as Table 6 for inclusive $p{{\bar{p}}} \rightarrow W^- + X \rightarrow l^- \nu + X$ production at $\sqrt{s}=1.96$ TeV

min $y_l$	cntr $y_l$	max $y_l$	$\sigma _{\textrm{LO}}$ [fb]	$\sigma _{\textrm{NLO}}$ [fb]	$\sigma _{\textrm{NNLO}}$ [fb]
− 3.2	− 2.95	− 2.7	7572 (0)	9013 (1)	9177 (2)
− 2.7	− 2.55	− 2.4	26,923 (1)	32,179 (3)	32,767 (6)
− 2.4	− 2.3	− 2.2	50,319 (2)	60,117 (5)	61,104 (16)
− 2.2	− 2.1	− 2.0	75,806 (3)	90,296 (6)	91,699 (22)
− 2.0	− 1.9	− 1.8	105,591 (3)	125,011 (7)	126,677 (29)
− 1.8	− 1.7	− 1.6	136,730 (3)	160,142 (9)	161,692 (35)
− 1.6	− 1.4	− 1.2	176,866 (3)	202,613 (6)	203,843 (22)
− 1.2	− 1.1	− 1.0	204,318 (4)	229,345 (11)	230,505 (47)
− 1.0	− 0.9	− 0.8	213,404 (4)	236,976 (11)	238,445 (47)
− 0.8	− 0.7	− 0.6	216,709 (4)	238,721 (11)	240,343 (45)
− 0.6	− 0.5	− 0.4	215,604 (4)	236,278 (11)	238,212 (44)
− 0.4	− 0.3	− 0.2	211,355 (4)	230,888 (10)	233,115 (45)
− 0.2	− 0.1	0.0	204,953 (4)	223,595 (10)	225,952 (44)
0.0	0.1	0.2	197,181 (4)	215,043 (10)	217,516 (42)
0.2	0.3	0.4	188,503 (4)	205,688 (9)	208,133 (39)
0.4	0.5	0.6	179,268 (4)	195,782 (9)	198,264 (37)
0.6	0.7	0.8	169,669 (4)	185,482 (9)	187,997 (37)
0.8	0.9	1.0	159,752 (3)	174,711 (8)	177,061 (36)
1.0	1.1	1.2	149,455 (3)	163,430 (8)	165,686 (35)
1.2	1.4	1.6	132,819 (2)	144,938 (5)	146,791 (17)
1.6	1.7	1.8	114,222 (3)	124,137 (7)	125,506 (34)
1.8	1.9	2.0	100,112 (3)	125,021 (7)	109,334 (30)
2.0	2.1	2.2	84,665 (3)	90,292 (7)	91,686 (29)
2.2	2.3	2.4	68,282 (2)	72,826 (7)	73,109 (28)
2.4	2.55	2.7	47,758 (1)	50,315 (4)	50,373 (17)
2.7	2.95	3.2	20,544 (1)	20,947 (2)	20,778 (7)

We also considered product cuts [22], which replace the separate linear asymmetric cuts on the leading and sub-leading lepton transverse momenta with cuts on their product and the sub-leading lepton instead. For values of $\Delta p_T \sim 10$ GeV these cuts mitigate the ambiguities of the perturbative series present with (a)symmetric cuts, which can be particularly relevant for processes with Casimir enhancement, notably Higgs production [63]. As expected, we find that their introduction does not address the pathological behavior for $\Delta p_T \rightarrow 0$, which can only be addressed by resummation. We found that resummation indeed removes this effect, although a kink in the cross section is still present for $\Delta p_T=0$, essentially due to the way we defined the observable for this study. At NNLO the differences induced by resummation are small for all the values of $\Delta p_T$ considered, ranging from 0 to a few GeV, shifting the fixed-order result by sub-percent values that are well below the current experimental uncertainties. An analogous consideration holds also at the level of the rapidity spectra, in agreement with the findings of Ref. [56]. It remains to be seen whether such conclusion still holds beyond NNLO, although recent studies [13, 22] indicate that the effect of resummation of linear power corrections remains moderate also at N$^3$LO. Given the excellent job done by the available fixed-order codes in reproducing the results of resummation and the substantial effort needed to implement the relevant modifications when modeling signal and background in experimental analyses, we foresee only a marginal improvement in adopting a different set of cuts in the Drell–Yan case for the measurement of fiducial cross sections.

Note added in proofs: The NNLOJET code has been published now in Ref. [64] and is available from https://nnlojet.hepforge.org/.

## Data Availability

This manuscript has no associated data. [Author’s’ comment: Data sharing not applicable to this article as no datasets were generated or analysed during the current study.]

## Appendix A: Cross section predictions

We present here the benchmark predictions discussed in Sect. 2 for the (pseudo-)rapidity distributions of the decay leptons for $W^\pm$- and $Z/\gamma ^*$-production at a center-of-mass energy of $\sqrt{s}=7$ TeV, corresponding to the fiducial cuts of the ATLAS experiment. The predictions listed here are all computed with NNLOJET and results are provided for each perturbative order (LO, NLO, NNLO). Note that all predictions use the same NNLO PDF set from ABMP16 [3].

We provide predictions for the differential distributions; the cross sections in columns 4, 5 and 6 of Tables 1, 2, 3 and 4 are already divided by the bin widths. Note also that we bin in the absolute value of the rapidities. This means the normalization is different by a factor of two compared to a calculation that bins the signed value of rapidity.

The predictions for the distributions in the electron pseudo-rapidity for the electron charge asymmetry measured by the DØ experiment in $W^\pm$-boson production at $\sqrt{s}=1.96$ TeV at the Tevatron [24]. The fiducial cuts and the settings are listed in Sect. 2. The predictions are computed with NNLOJET, use the same NNLO PDF set from ABMP16 [3] for each perturbative order (LO, NLO, NNLO) and are again provided for the differential distributions, i.e., the cross sections (columns 4, 5 and 6 in Tables 5, 6) are already divided by the bin widths.

## Appendix B: DYTURBO inputs

The input values used for the computations with DYTURBO for *Z*-boson production with central leptons at $\sqrt{s}=7$ TeV in Sect. 2.

## Appendix C: FEWZ runcard

The runcard used for the computations with FEWZ (version 3.1) for *Z*-boson production with central leptons at $\sqrt{s}=7$ TeV in Sect. 2.

## Appendix D: MATRIX runcard

The runcard used for the computations with MATRIX (version 2.1) for *Z*-boson production with central leptons at $\sqrt{s}=7$ TeV in Sect. 2.

## Appendix E: Set-up for SCETlib

The relevant physical settings and cuts on $Q \equiv m_{ll}  { and}Y \equiv Y_{l l}$ used for the matched predictions in Sect. 4 are given by the SCETlib input file below. The cuts on the lepton transverse momenta are described in the main text. Note that we always cut on the lepton pseudorapidities $\eta _{l_1,l_2} \le 2.5$ in addition.

## Footnotes

1 Code available from https://dyturbo.hepforge.org/.

2 Code available from https://www.hep.anl.gov/fpetriello/FEWZ.html.

3 Code available from https://matrix.hepforge.org/.

4 Private code. Results provided from the authors upon request.

5 The DYNNLO program, considered in the previous comparison [17], is a legacy code now superseded by DYTurbo.

6 Code available from https://mcfm.fnal.gov/.

7 The impact of the fiducial cuts applied to $Z/\gamma ^*$-boson production for different values of the gauge-boson rapidity $y_{ll}$ on the real emission phase space of the decay leptons has been illustrated in Fig. 15 of Ref. [17].

## Code Availability Statement

This manuscript has no associated code/software. [Author’s comment: Code/Software sharing not applicable to this article as no code/software was generated or analysed during the current study.]

## References

[1] ATLAS, G. Aad et al., Eur. Phys. J. C **80**, 616 (2020). arXiv:1912.02844

[2] CMS, A.M. Sirunyan et al., JHEP **12**, 059 (2019). arXiv:1812.10529

[3] S. Alekhin, J. Blümlein, S. Moch, R. Placakyte, Phys. Rev. D **96**, 014011 (2017). arXiv:1701.05838

[4] S. Bailey et al., Eur. Phys. J. C **81**, 341 (2021). arXiv:2012.04684

[5] NNPDF, R.D. Ball et al., Eur. Phys. J. C **78**, 408 (2018). arXiv:1802.03398 10.1140/epjc/s10052-018-5897-7 PMC6435224 30996667

[6] ATLAS, M. Aaboud et al., Eur. Phys. J. C **78**, 110 (2018). arXiv:1701.07240 [Erratum: Eur. Phys. J. C 78, 898 (2018)]

[7] LHCb, R. Aaij et al., JHEP **01**, 036 (2022). arXiv:2109.01113

[8] C. Duhr, F. Dulat, B. Mistlberger, JHEP **11**, 143 (2020). arXiv:2007.13313

[9] C. Duhr, B. Mistlberger, JHEP **03**, 116 (2022). arXiv:2111.10379

[10] S. Camarda, L. Cieri, G. Ferrera, Phys. Rev. D **104**, L111503 (2021). arXiv:2103.04974

[11] E. Re, L. Rottoli, P. Torrielli, (2021). arXiv:2104.07509

[12] W.L. Ju, M. Schönherr, JHEP **10**, 088 (2021). arXiv:2106.11260

[13] X. Chen et al., Phys. Rev. Lett. **128**, 252001 (2022). arXiv:2203.01565 35802442 10.1103/PhysRevLett.128.252001

[14] T. Neumann, J. Campbell, (2022). arXiv:2207.07056

[15] S. Camarda, L. Cieri, G. Ferrera, Phys. Lett. B **845**, 138125 (2023). arXiv:2303.12781

[16] S. Catani, M. Grazzini, Phys. Rev. Lett. **98**, 222002 (2007). arXiv:hep-ph/0703012 17677837 10.1103/PhysRevLett.98.222002

[17] S. Alekhin, A. Kardos, S. Moch, Z. Trócsányi, Eur. Phys. J. C **81**, 573 (2021). arXiv:2104.02400 10.1140/epjc/s10052-025-14027-x PMC11985598 40224926

[18] S. Catani et al., Phys. Rev. Lett. **103**, 082001 (2009). arXiv:0903.2120 19792718 10.1103/PhysRevLett.103.082001

[19] R. Gavin, Y. Li, F. Petriello, S. Quackenbush, Comput. Phys. Commun. **184**, 208 (2013). arXiv:1201.5896

[20] R. Boughezal, C. Focke, X. Liu, F. Petriello, Phys. Rev. Lett. **115**, 062002 (2015). arXiv:1504.02131 26296111 10.1103/PhysRevLett.115.062002

[21] S. Frixione, G. Ridolfi, Nucl. Phys. B **507**, 315 (1997). arXiv:hep-ph/9707345

[22] G.P. Salam, E. Slade, JHEP **11**, 220 (2021). arXiv:2106.08329

[23] ATLAS, M. Aaboud et al., Eur. Phys. J. C **77**, 367 (2017). arXiv:1612.03016 10.1140/epjc/s10052-017-4911-9 PMC6129393 30215626

[24] D0, V.M. Abazov et al., Phys. Rev. D **91**, 032007 (2015). arXiv:1412.2862. [Erratum: Phys. Rev. D 91, 079901 (2015)]

[25] S. Dittmaier, M. Huber, JHEP **01**, 060 (2010). arXiv:0911.2329

[26] Particle Data Group, P.A. Zyla et al., Prog. Theor. Exp. Phys. **2020**, 083C01 (2020)

[27] S. Alekhin, J. Blümlein, S. Moch, Eur. Phys. J. C **78**, 477 (2018). arXiv:1803.07537

[28] S. Camarda et al., Eur. Phys. J. C **80**, 251 (2020). arXiv:1910.07049. [Erratum: Eur. Phys. J. C 80, 440 (2020)]

[29] Y. Li, F. Petriello, Phys. Rev. D **86**, 094034 (2012). arXiv:1208.5967

[30] L. Buonocore, S. Kallweit, L. Rottoli, M. Wiesemann, Phys. Lett. B **829**, 137118 (2022). arXiv:2111.13661

[31] F. Cascioli, P. Maierhofer, S. Pozzorini, Phys. Rev. Lett. **108**, 111601 (2012). arXiv:1111.5206 22540459 10.1103/PhysRevLett.108.111601

[32] M. Grazzini, S. Kallweit, M. Wiesemann, Eur. Phys. J. C **78**, 537 (2018). arXiv:1711.06631

[33] A. Gehrmann-De Ridder et al., JHEP **05**, 002 (2023). arXiv:2301.11827

[34] C. Anastasiou, K. Melnikov, F. Petriello, Phys. Rev. D **69**, 076010 (2004). arXiv:hep-ph/0311311 10.1103/PhysRevLett.93.032002 15323816

[35] A. Gehrmann-De Ridder, T. Gehrmann, E.W.N. Glover, JHEP **09**, 056 (2005). arXiv:hep-ph/0505111

[36] R. Gauld et al., PoS **RADCOR2019**, 002 (2019)

[37] M. Grazzini et al., JHEP **08**, 140 (2016). arXiv:1605.02716

[38] M.A. Ebert et al., JHEP **04**, 123 (2019). arXiv:1812.08189

[39] L. Buonocore, M. Grazzini, F. Tramontano, Eur. Phys. J. C **80**, 254 (2020). arXiv:1911.10166 32226282 10.1140/epjc/s10052-020-7815-z PMC7089628

[40] L. Cieri, C. Oleari, M. Rocco, Eur. Phys. J. C **79**, 852 (2019). arXiv:1906.09044

[41] C. Oleari, M. Rocco, Eur. Phys. J. C **81**, 183 (2021). arXiv:2012.10538

[42] M.A. Ebert, F.J. Tackmann, JHEP **03**, 158 (2020). arXiv:1911.08486

[43] S. Catani, D. de Florian, G. Ferrera, M. Grazzini, JHEP **12**, 047 (2015). arXiv:1507.06937

[44] M. Grazzini, S. Kallweit, D. Rathlev, M. Wiesemann, JHEP **08**, 154 (2015). arXiv:1507.02565

[45] M.A. Ebert, J. Michel, I.W. Stewart, F.J. Tackmann, JHEP **04**, 102 (2021). arXiv:2006.11382

[46] J. Campbell, T. Neumann, JHEP **12**, 034 (2019). arXiv:1909.09117

[47] R. Boughezal et al., Eur. Phys. J. C **77**, 7 (2017). arXiv:1605.08011

[48] J. Gaunt, M. Stahlhofen, F.J. Tackmann, J.R. Walsh, JHEP **09**, 058 (2015). arXiv:1505.04794

[49] J. Campbell, T. Neumann, JHEP **11**, 127 (2023). arXiv:2308.15382

[50] J. Campbell, T. Neumann, G. Vita, arXiv:2408.05265

[51] X. Chen, T. Gehrmann, E.W.N. Glover, A. Huss, B. Mistlberger, A. Pelloni, Phys. Rev. Lett. **127**(7), 072002 (2021). arXiv:2102.07607 10.1103/PhysRevLett.127.072002 34459639

[52] A. Bhattacharya et al., JHEP **11**, 080 (2023). arXiv:2306.08033

[53] G. Lustermans, J. Michel, F.J. Tackmann, W.J. Waalewijn, JHEP **03**, 124 (2019). arXiv:1901.03331

[54] I.W. Stewart, F.J. Tackmann, J.R. Walsh, S. Zuberi, Phys. Rev. D **89**(5), 054001 (2014). arXiv:1307.1808

[55] D. Bertolini, M.P. Solon, J.R. Walsh, Phys. Rev. D **95**(5), 054024 (2017). arXiv:1701.07919

[56] S. Amoroso et al., (2022). arXiv:2209.13535

[57] ATLAS, M. Aaboud et al., JINST **14**, P03017 (2019). arXiv:1812.03848

[58] ATLAS, G. Aad et al., Eur. Phys. J. C **83**, 686 (2023). arXiv:2212.07338

[59] CMS, A.M. Sirunyan et al., Phys. Rev. D **102**, 092012 (2020). arXiv:2008.04174

[60] CMS, S. Chatrchyan et al., JHEP **10**, 007 (2011). arXiv:1108.0566

[61] ATLAS, G. Aad et al., Phys. Lett. B **759**, 601 (2016). arXiv:1603.09222

[62] ATLAS, G. Aad et al., Eur. Phys. J. C **83**, 982 (2023). arXiv:2212.09379

[63] G. Billis et al., Phys. Rev. Lett. **127**, 072001 (2021). arXiv:2102.08039 34459622 10.1103/PhysRevLett.127.072001

[64] A. Huss *et al.* [NNLOJET], (2025), arXiv:2503.22804

